# Mitigating disease risk in an endangered pinniped: early hookworm elimination optimizes the growth and health of Australian sea lion pups

**DOI:** 10.3389/fvets.2023.1161185

**Published:** 2023-04-25

**Authors:** Scott A. Lindsay, Mariel Fulham, Charles G. B. Caraguel, Rachael Gray

**Affiliations:** ^1^Faculty of Science, Sydney School of Veterinary Science, The University of Sydney, Camperdown, NSW, Australia; ^2^Faculty of Science, School of Animal and Veterinary Sciences, The University of Adelaide, Roseworthy, SA, Australia

**Keywords:** Australian sea lion, growth, hematology, hookworm, ivermectin, *Neophoca cinerea*, otariid, *Uncinaria sanguinis*

## Abstract

The Australian sea lion (*Neophoca cinerea*) experiences high pup mortality of seasonally alternating severity, partly attributed to endemic hookworm (*Uncinaria sanguinis*) infection. To further explore health outcomes of early hookworm elimination, a treatment trial was conducted at Seal Bay Conservation Park, South Australia, over consecutive lower and higher mortality breeding seasons (2019, 19.2%; 2020–1; 28.9%). Pups (*n* = 322) were stratified into two age cohorts (median 14 d and 24 d recruitment ages) and randomly assigned to treated (topical ivermectin 500  μg/kg) or control (untreated) groups. A younger prepatent cohort <14 d old (median 10 d) was identified *a posteriori*. A seasonally independent growth benefit resulted from hookworm elimination across all age cohorts. The greatest relative improvements (bodyweight + 34.2%, standard length + 42.1%; *p* ≤ 0.001) occurred in the month post-treatment, in the youngest prepatent cohort. A significant benefit of lesser magnitude (bodyweight + 8.6–11.6%, standard length + 9.5–18.4%; *p* ≤ 0.033) persisted up to 3  months across all age cohorts – greatest in the youngest pups. Treatment resulted in immediate improvement in hematological measures of health – decreased anemia and inflammation severity (*p* ≤ 0.012). These results enhance our understanding of host–parasite–environment interactions within the context of hematological ontogenesis, confirm the seasonally independent benefits of hookworm disease intervention, and further inform conservation recommendations for this endangered species.

## 1. Introduction

The contribution of infectious disease to wildlife species’ decline is likely underestimated due to limitations in population monitoring, detection of diseased individuals, and in assigning etiology [reviewed in (1)]. This holds true for pinnipeds (seals and sea lions), which spend a substantial amount of time foraging at sea and haul out on remote coastlines, such that conclusive evidence of infectious disease compromising their populations is often relatively limited (2, 3). For populations exposed to endemic infections there is a ubiquitous risk of a disease outbreak should additional stressors alter the host-pathogen-environment balance (1, 4, 5). Equally, endemic infection can compromise host resilience against evolving population stressors, such as those attributed to climate change – temperature extremes and altered foraging ranges (1, 6, 7). Consequently, deconstructing factors determining endemic infection elaboration and the success of mitigation strategies, is essential for enhanced conservation medicine (8, 9).

Disease impact on population sustainability is particularly relevant for an endangered and declining (10) species such as the Australian sea lion (*Neophoca cinerea*). The species’ ability to maintain its population in the presence of increased disease pressure is potentially limited by (1) the absence of a population buffer – less than 10,000 individuals, and (2) a segmented population structure – 80 dispersed breeding colonies (11), each representing a genetically contracted subpopulation with limited adult female dispersal capacity due to extreme fine-scale foraging and philopatric behavior (12, 13). Further, the species’ fecundity is low because of female reproduction commencing at 4.5–6.0 years of age, extended non-annual breeding seasons 17–18-months apart, and high mortality among the 2,700 pups born each breeding season (14–17). Ultimately, pup mortality is the major contributor to individuals not reaching breeding age – a progressive increase in age-specific survival apparent in older cohorts, reaching a plateau in early adulthood (18). A high proportion of pup deaths occur in the first few weeks of life, most often attributed to conspecific trauma, starvation (19), and the direct and indirect impact of endemic hookworm (*Uncinaria sanguinis*) infection (20). Consequently, pup disease and specifically hookworm infection, is listed as one of the key threats to the species’ recovery (10).

*Uncinaria sanguinis* infection occurs in all Australian sea lion pups (20). Their single point of infection is ingestion of infective larvae in colostrum (20) – the milk produced within the first 24–48 h after birth. Detection of parasite ova in pup feces (patency) from 11 to 14 days of age confirms larval maturation to adult hookworms in the intestines and their elimination occurs by 2–3 months of age (post-patency) (20). Hookworm is a compromising agent of Australian sea lion pups, its blood-sucking activity contributing to intestinal loss of red blood cells and protein and to local and systemic inflammation (eosinophilic and lymphocytic) (21, 22). Most pup deaths directly attributable to infection occur from hemorrhagic enteritis in the first few weeks of life, supporting a pathogenic contribution from juvenile hookworms in the prepatent period, as well as from adult hookworms (20, 23). Additionally, infection contributes indirectly to pup deaths from increased conspecific trauma in disease-weakened pups, supported by reporting of infection intensity-dependent mortality (20). Two anti-parasiticide treatment trials at Dangerous Reef, Spencer Gulf, South Australia have studied the short-term health benefits of altering the host-parasite relationship in this species. The first trial successfully used an injected anthelmintic (ivermectin) to eliminate hookworm infection and to reduce the severity of anemia (reduction in red blood cell concentration) and systemic eosinophilic inflammation (24). The second trial demonstrated an equivalent effectiveness of a less invasive topical (“spot-on”) formulation of ivermectin in comparison to the injectable formulation and revealed a treatment benefit for the short-term loss in body condition associated with hookworm infection (21). These studies were precautious in recruitment size and duration, partly to enable assessment of any adverse effects of ivermectin on the host and partly due to challenging site logistics. In related otariid (eared seal) species, longer term benefits of early hookworm elimination have included increased pup growth, improved pup health based on the evaluation of hematological parameters, and ultimately increased pup survival – the latter most often following treatment of the youngest pups (<2 weeks old) during breeding seasons of higher than usual mortality (caused either by hookworm or an unrelated comorbidity) (25, 26).

Australian sea lion pup mortality oscillates between breeding seasons (14, 15, 27, 28). At the Seal Bay colony on Kangaroo Island, South Australia, higher pup mortality is observed during summer–autumn (versus winter–spring) breeding seasons (15). Reasons for this variation are not fully understood. Proposed contributions include anomalies in sea surface temperature impacting maternal provisioning of pups (29), direct environmental challenge (temperature and weather extremes), higher population density leading to increased conspecific trauma (27), and endemic or emerging disease (20). Significant (*p* < 0.05) correlations exist between hookworm infection intensity, pup mortality, and season at the Seal Bay colony (20). While this does not necessarily equate to causation, hookworm burden perhaps reflecting another comorbidity or population stressor, it highlights the need to consider seasonal impact in any assessment of hookworm disease outcome or its modulation. In the South American fur seal (*Arctocephalus australis*), seasonal variability in pup ability to mount an immune response to eliminate hookworm infection has been attributed to impacts on maternal provisioning of pups (30).

This study investigated the long-term benefit of topical ivermectin on pup health by conducting a large-scale randomized controlled field trial with extended three-month follow-up period over two consecutive breeding seasons at the Seal Bay colony. In addition to treatment effectiveness in clearing hookworm infection, we investigated the optimization of health benefits reflected in pup growth and hematological profiles, by analyzing outcomes in different age cohorts at treatment delivery.

## 2. Materials and methods

### 2.1. Ethics approvals and reporting

All samples were collected with the approval of the South Australian Department of Environment and Water (Permit/License Number: MR00073-2-R; Scientific Research Permit Number: A25088-12) and the trial protocol was approved by The University of Sydney Animal Ethics Committee (Protocol Number 2017/1260). Recommendations in the CONSORT (Consolidated Standards of Reporting Trials) statement were used to guide reporting of the study outcomes (31).

### 2.2. Study site and duration

The study was conducted at Seal Bay Conservation Park, Kangaroo Island, South Australia (−35.996, 137.327), the third largest breeding colony of Australian sea lions. The long-term trend (12 season average) shows a 1.3% decline in seasonal pup production, with recent stabilization (five seasons) (14). During extended 7–12 month breeding seasons, twice weekly colony monitoring provides an accurate assessment of pup births (accurate individual age for some pups) and deaths (14), and routine monthly microchip scanning and animal counts provide temporal information at an individual and colony level, respectively. Routine pup microchipping offers permanent and unique animal identification, enabling longitudinal observation of individuals and mother-pup pairing. Indicative intra-seasonal pup mortality incidence over the previous 11 breeding seasons were 21.4% (range 17.9–24.7%) for winter–spring seasons and 32.8% (range 28.0–41.8%) for summer–autumn seasons (14). The present trial was conducted in the lower mortality 2019 breeding season (estimated 239 births, 46 deaths and 19.2% mortality incidence) and the higher mortality 2020–1 summer-autumn breeding season (estimated 242 births, 70 deaths and 28.9% mortality incidence) (14).

### 2.3. Study design and sample collection

A randomized controlled field-based treatment trial initially recruited young Australian sea lion pups <80 cm in standard length (snout-to-tail-tip length; an age proxy) to one of two age cohorts – a younger <70 cm cohort and an older ≥70 cm cohort. After preliminary analysis of season 2019 data the study design was refined (1) to a 75 cm upper length restriction to limit the recruitment of postpatent pups (>60 d of age), and (2) by increasing the frequency of colony observation to detect pups earlier and thereby maximize recruitment to the younger cohort. Within each age cohort, pups were randomly assigned one of two treatment groups – an untreated control group or a topical anthelmintic treated group. Treatment allocation was concealed, but not blinded, from treatment providers until the time of potential anthelmintic application and blinded from handlers at pup recaptures and from operators during sample processing. Predetermined randomization within each age cohort was achieved as previously described (21), with addition of blocking in groups of four in season 2020–1 to maintain equal pup numbers within each treatment group. Pups were recruited each season at all but the final colony visit and recaptured when possible, up to three times at subsequent monthly visits. To assess treatment impact on the youngest of recruited pups prior to the development of patent infection, a third prepatent age cohort was identified *a posteriori*, which comprised pups <14 d of age with a negative fecal hookworm test at recruitment (hereafter referred to as the <14 d and youngest cohort). No distinct random allocation was applied to this cohort.

Protocols for pup health assessment, sample collection and treatment delivery were conducted as previously described (21). Briefly, pup health and morphometric data included sex, bodyweight, standard length, subjective four-scale body condition score, lice infestation, and a visual lice infestation intensity score (lice score: 0–9). Recorded capture details included fieldtrip order (sequential numbering of colony visits to assess for impact from intraseasonal climatic variation), date, and initial capture state [reported as sleeping, awake or mobile, and subsequently used to assess for variable impact of capture stress on hematological parameters as previously identified (22)]. Pups were individually identified by a unique hair clip and bleach pattern (persisting until the first molt at 4–5 months of age), and by microchipping of pups weighing approximately 10 kg or more (HDX, 23 mm; Allflex Australia Pty Ltd., Capalaba, Qld). Fecal swab and EDTA-anticoagulated peripheral blood samples collected from each pup were chilled to approximately 4°C in the field. Treated pups received topical ivermectin based on bodyweight (IVOMEC® Pour-on for Cattle, 5 mg/ml, Boehringer Ingelheim Animal Health Australia Pty. Ltd., North Ryde NSW; 500 μg/kg; off-label use). Control pups underwent identical handling and assessment to treated pups, except for anthelmintic treatment.

### 2.4. Specimen processing

#### 2.4.1. Blood analysis

Packed cell volume (PCV; L/L) and total plasma protein (TPP; g/L) concentration were manually determined on the day of sampling, while automated hematological analysis (Sysmex XT-2000iV, Sysmex, Kobe, Japan) of preserved blood specimens (Streck Cell Preservative; Streck, Omaha, USA) was performed 2–8 days later (21). Automated analysis provided indicators of red blood cell number additional to PCV (red blood cell [RBC; ×10^12/L] count and hemoglobin [Hb; g/L] concentration), as well as platelet (PLT; ×10^9/L) and total white blood cell (WBC; ×10^9/L) counts. Duplicate Wrights-Giemsa-stained blood smears prepared the day of sampling were later reviewed for manual differential WBC and nucleated RBC (nRBC) counts. Reticulocyte percentage was determined from new methylene blue-stained blood smear counts (Reticulocyte stain, Sigma-Aldrich, Germany) using a Miller Square (9:1 ratio) eyepiece reticule to count the equivalent of >1,000 RBC. In addition to nRBC, the absolute reticulocyte count for each pup was subsequently calculated (reticulocyte percentage x RBC count) as a measure of the intensity of RBC regeneration.

#### 2.4.2. Fecal analysis

The presence of a single *U. sanguinis* ovum [elliptical, thin shelled, 110–135 μm × 55–95 μm; (23)] in any glass slide smear (minimum of two reviewed) prepared from each fecal swab was sufficient for reporting positive patent hookworm infection (21). Hookworm incidence in Seal Bay pups has previously been reported as 100% (22). Consequently, a negative fecal smear at recruitment was interpreted as prepatent infection (with pup age reviewed [<60 days] to exclude the likelihood of postpatent infection), and at any of the recaptures as postpatent infection (with review of subsequent capture results [all negative] to exclude false negative results).

### 2.5. Statistical analysis

Statistical analysis was performed using version 17.0 of the Stata statistical package (StataCorp LLC., College Station, Texas, United States) and results were considered significant at the 5% level (type-I-error or α). The study utilised standard multifactorial modelling to compare treatment groups, unless otherwise described. A base additive model included by default treatment allocation as the primary factor of interest, season, and a set of biological parameters (outlined below) expected to predict the model outcome (i.e., biological hypothesis). All base additive predictors were retained in the final model, irrespective of significance. Additional ancillary factors were then explored for significance one at the time in addition to the base additive model, and only those significantly associated were considered for inclusion into the final model. For strongly collinear predictors, only the most biologically meaningful factor was retained. All possible pairwise (first order) interactions were explored among the remaining factors and retained in the final model if significant. Pup age was available for pups with a birth date record that were able to be monitored for identification until the time of recruitment (possible due to limited colony movement of neonatal pups). For pups without a birth date, an estimated age at recruitment was predicted from a multifactorial linear regression model using records from pups of known age (refer to Supplementary Data S1). Pup age at recaptures was calculated by adding the known period length (days) between captures to the age at recruitment.

#### 2.5.1. Random allocation

To evaluate successful random allocation, pup demographics and hematological parameters at recruitment were compared between the two treatment groups, in the <70 and ≥70 cm age cohorts, as well as in the prepatent <14 d old pup cohort which was largely a subset of the <70 cm cohort with fewer individuals in the ≥70 cm cohort. For continuous outcomes with no evidence of deviation from normality, group means were reported and compared using the non-paired t-test. When a continuous outcome revealed strong deviation from normality, a non-parametric Wilcoxon rank-sum test was used to test equality of group distribution and medians were reported. For binary outcomes, frequencies within treatment groups were compared using Fisher’s exact test.

#### 2.5.2. Effectiveness

Topical ivermectin effectiveness was estimated by comparing either the predicted prevalence of patent hookworm infection at first recapture or the predicted incidence of hookworm clearance in the recruitment-to-first recapture period in both treatment groups, to calculate relative risk reductions (21). The incidence of hookworm clearance was calculated as the complement of the predicted proportion of pups with patent infection at recruitment that remained patent at recapture. Using Firth’s variant of the multifactorial logistic regression, the base additive model included treatment group, season, and pup age at first recapture. Ancillary factors assessed for significance were sex; bodyweight, standard length, and body mass index [BMI; included for consistency with previous reporting (21)] at first recapture; first recapture fieldtrip; and the recruitment-to-first recapture period length.

#### 2.5.3. Growth

The treatment effect on pup growth was assessed by calculating a daily specific growth rate (SGR) for bodyweight and standard length, for the periods between each capture and for the period from recruitment to final (third) recapture (21). The base additive model included treatment group, season, pup age and baseline growth parameter result at the start of the period, and the period length. Additional ancillary factors assessed and retained if found to have a significant effect on the daily SGR estimate, were sex; BMI, the alternative growth parameter, fieldtrip, and patent hookworm presence at the start of the period; and lice presence at the start and end of the period.

#### 2.5.4. Hematology

When evaluating treatment effect on hematological parameters, data distribution and presence of potential outliers for each analyte were explored visually by a scatterplot of analyte value against standard length, a histogram of analyte results, and the interquartile range (IQR) rule. For RBC and platelet results, the latter used three times the IQR to identify extreme outliers. These were subsequently reviewed and excluded if *in vivo* or *in vitro* sample hemolysis artefact was suggested by the reported sample appearance at collection, or by a comparatively large discrepancy between the manually determined PCV (assessed the day of collection) and the automated hematocrit (assessed 2–8 days later). For the WBC parameters, which were less impacted by sample artefact, four times the IQR was used to identify extreme outliers for exclusion. Means for each hematological analyte at each recapture were compared between treatment groups for each of the age cohorts. The base additive model included treatment group, season, pup age at current capture and the analyte measure at the previous capture (for pup recaptures – a baseline analyte level). Additional ancillary factors included sex, the presence of lice, the presence of patent hookworm, standard length, bodyweight, BMI, fieldtrip, and capture state at current capture. To satisfy the assumptions of a linear regression, a Box-Cox analysis was performed on the maximized model to detect the need for outcome value transformation. All transformed model estimates and their associated 95% CI boundaries were back transformed prior to reporting (these estimates should be interpreted as medians).

### 2.6. Survival

Analysis of potential association of treatment with pup survival and cause of mortality will be reported elsewhere.

## 3. Results

### 3.1. Recruitment, success of random allocation and recapture rates

**Figure 1:**
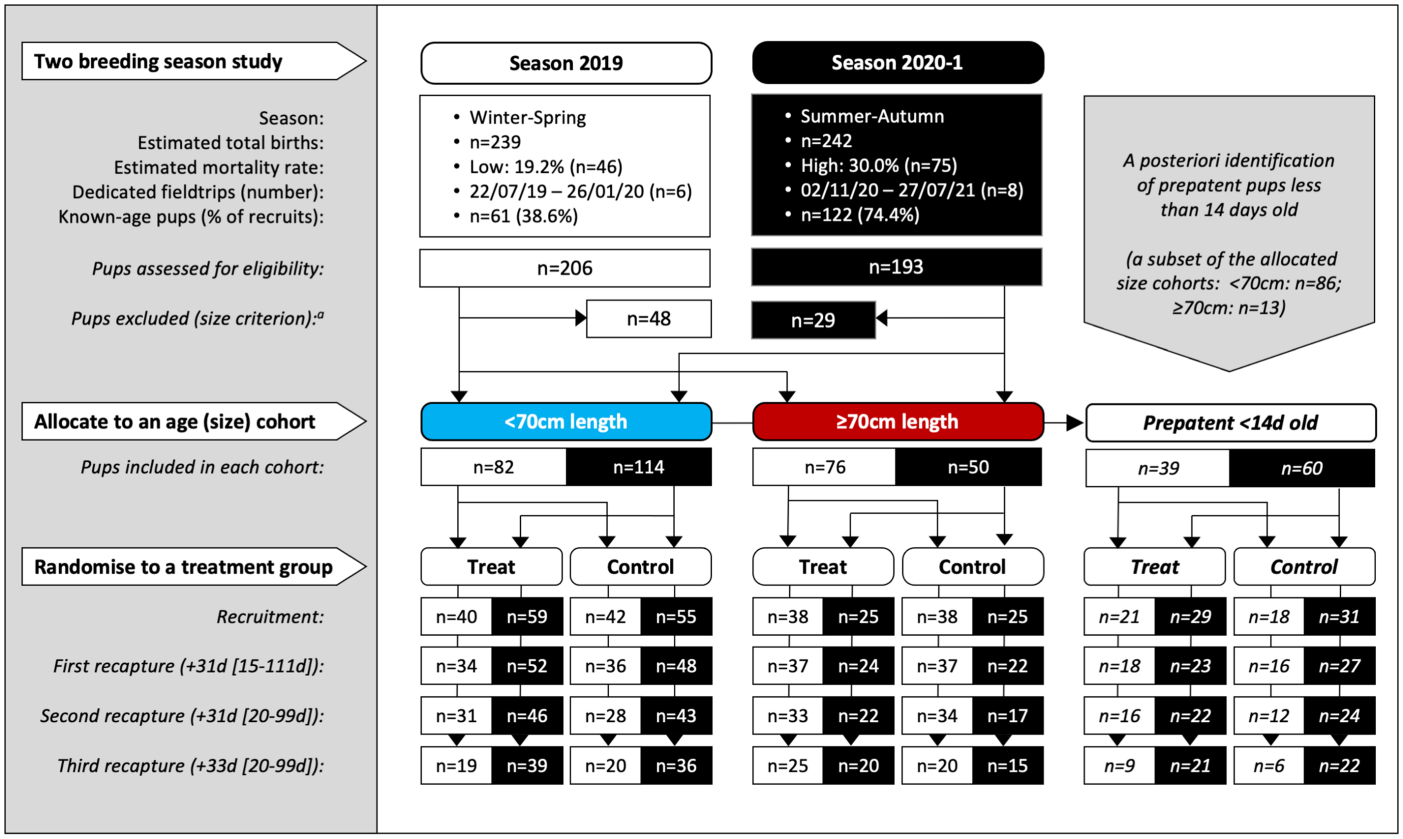
Study design for the two-season, randomized and controlled field-based trial of Australian sea lion pups at Seal Bay Conservation Park, Kangaroo Island, South Australia. Pups were recruited in consecutive breeding seasons (at all fieldtrips except the last of each season) and recaptured up to three times at subsequent monthly fieldtrips within the same season. Within two age cohorts (allocated by the standard length proxy to <70 and ≥70 cm) pups were randomized to treatment or control groups. A third cohort of prepatent pups <14 d old at recruitment was identified *a posteriori*. The median period length [and range] in days prior to recapture is shown for the combined age cohorts. ^a^Sixteen pups (*n* = 15, 2019; *n* = 1, 2020–1) >75 cm in standard length were recruited prior to implementing the 75  cm upper size restriction and were retained in the analysis.

**Figure 2:**
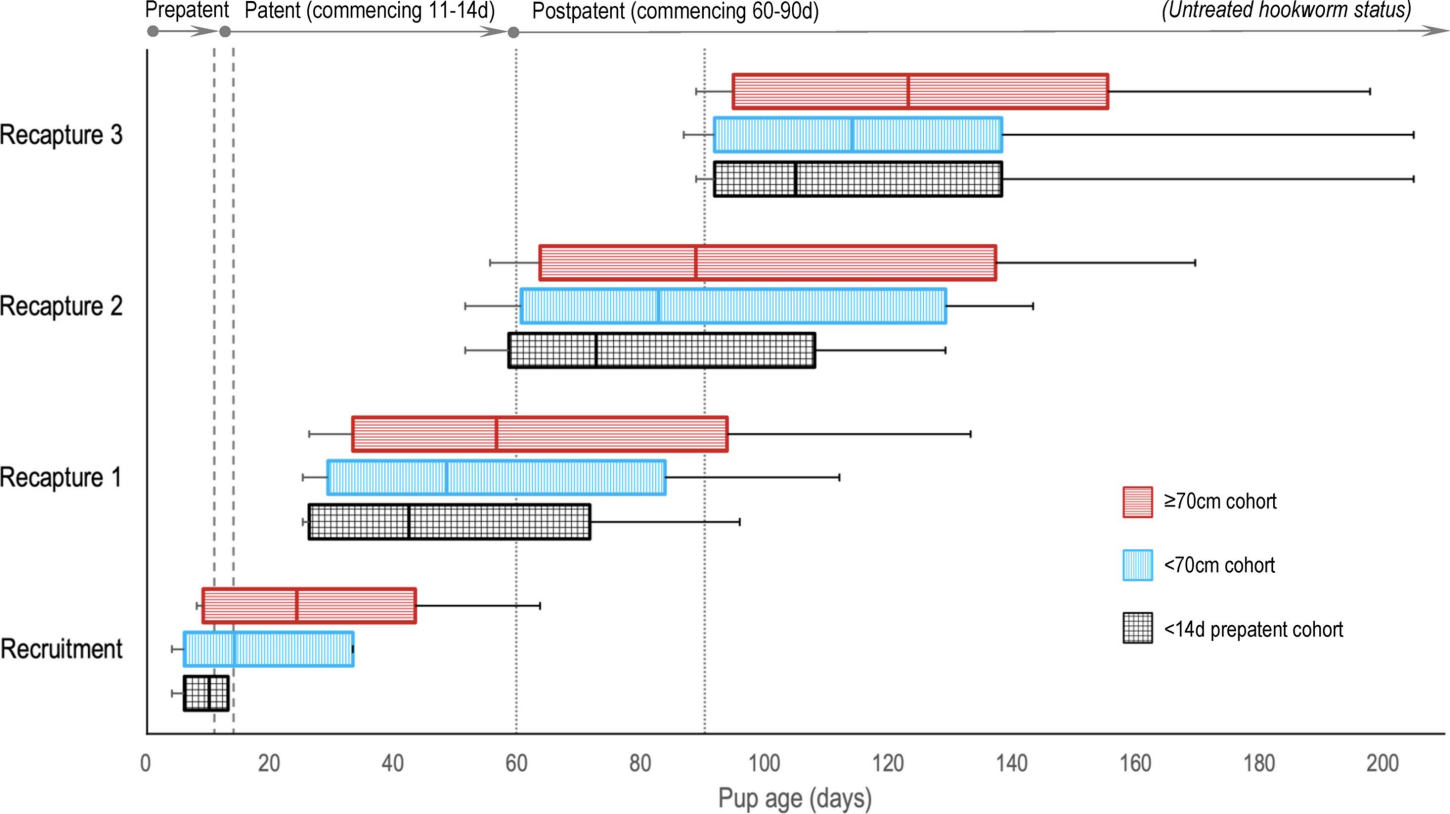
A comparison of Australian sea lion pup ages at recruitment and recaptures for the three age cohorts, relative to anticipated hookworm status in untreated pups. Patent hookworm infection develops from approximately 11–14 d of age (patency onset range – indicated by the vertical dashed lines), and infection is naturally cleared from approximately 60–90 d of age (patency termination range – indicated by the vertical dotted lines) (20). Boxes indicate the interquartile range for pup age with the median value marked (most distributions were right skewed), and the horizontal lines represent pup age range (minimum to maximum).

There were 14 dedicated recruitment fieldtrips across seasons 2019 and 2020–1 (*n* = 6, 2019; *n* = 8, 2020–1), resulting in 399 individual pups being assessed for eligibility (*n* = 206, 2019; *n* = 193, 2020–1; Figure 1). Of these, 322 (*n* = 158, 2019; *n* = 164, 2020–1) pups were recruited based on the pre-defined size criteria – 196 (*n* = 82, 2019; *n* = 114, 2020–1) into the <70 cm cohort and 126 (*n* = 76, 2019; *n* = 50, 2020–1) into the ≥70 cm cohort. Of these, 99 pups (*n* = 39, 2019; *n* = 60, 2020–1) were additionally subclassified into the third <14 d cohort. As expected, this age cohort was predominantly a subset of the younger <70 cm cohort (*n* = 86, <70 cm; *n* = 13, ≥70 cm). Sixteen pups (*n* = 15, 2019; *n* = 1, 2020–1) >75 cm in standard length were recruited prior to implementing the 75 cm upper size restriction and were retained in the analysis. Pup age at recruitment was known for 56.8% (n = 183) of the pups (38.6% [*n* = 61], 2019; 74.4% [*n* = 122], 2020–1), with age estimated for the remaining pups – median recruitment ages (and range) for the <14 d, <70 cm and ≥70 cm cohorts being 10 d (4–13 d), 14 d (4–33 d), and 24 d (8–63 d), respectively. Figure 2 shows pup age at recruitment and each recapture for each cohort, relative to the anticipated hookworm status in untreated pups – patency developing from 11 to 14 d and infection cleared from 60 to 90 d (20) (refer to Supplementary Table S1 for cohort age values at each recapture). Observed variation from these expectations based on fecal analysis (*n* = 381) in untreated control pups of known age were one prepatent pup 18 d old and six patent pups aged 8–10 d old at recruitment; two postpatent pups 40 d and 49 d old at recapture 1; six patent pups aged 92–124 d at recapture 2; and five patent pups aged 97–148 d at recapture 3. A total of 162 (*n* = 78, 2019; *n* = 84, early 2020–1) pups were allocated to receive the topical ivermectin treatment; 160 pups (*n* = 80, 2019; *n* = 80, early 2020–1) received no treatment.

Pup recruitment season, demographics and parasitic burden data are reported in Table 1, and pup recruitment hematology values are reported in Table 2. At recruitment, there were no significant differences for season, sex, age and size measures, and parasitic burdens, nor for any of the hematological variables, between treatment groups within any of the age cohorts (including within the <14 d cohort; *p* ≥ 0.05; Tables 1, 2). This is consistent with successful random allocation at recruitment. Median period length [and range] for the combined age cohorts for recruitment-to-first recapture (31 d [15–111 d]), first recapture-to-second recapture (31 d [20–99 d]), second recapture-to-final recapture (33 d [20–99 d]) and recruitment-to-final recapture (95 d [76–195 d]) periods was similar for each of the individual age cohorts. Excluding dead pups, the proportion of pups with a first, second and third recapture for both seasons combined were 96% (*n* = 291), 87% (*n* = 254), and 66% (*n* = 194), respectively. Where patent hookworm status was determined after death, five of 10 control pups and one of seven treated pups were positive. The positive treated pup (post-mortem smears totaling two hookworm ova over three slides) was found dead from conspecific trauma 3 days post-recruitment (recruitment smears >30 hookworm ova per slide), with heavy scavenging and marked post-mortem autolysis consistent with death >1–2 days earlier. No deaths in any of the treated pups were attributable to the topical ivermectin application.

**Table 1:** Recruitment season, demographics, and health details for treatment groups of Australian sea lion pups for each age cohort.

	Prepatent pups < 14 d old (*n* = 99)				Pups < 70 cm standard length (*n* = 196)				Pups ≥ 70 cm standard length (*n* = 126)			
Variable	Control		Treatment		Control		Treatment		Control		Treatment	
	*n*	Estimate	*n*	Estimate	*n*	Estimate	*n*	Estimate	*n*	Estimate	*n*	Estimate
Recruitment season												
2019	18	36.7%	21	42.0%	42	43.3%	40	40.4%	38	60.3%	38	60.3%
2020–1	31	63.3%	29	58.0%	55	56.7%	59	59.6%	25	39.7%	25	39.7%
Sex												
Male	24	49.0%	33	66.0%	40	41.2%	47	47.5%	37	58.7%	41	65.1%
Female	25	51.0%	17	34.0%	57	58.8%	52	52.5%	26	41.3%	22	34.9%
Age and size measures												
Age – median (range) days^a^^,^^b^	49	10 (4–13)	50	10 (5–13)	97	14 (4–33)	99	13 (5–33)	63	24 (9–63)	63	24 (8–47)
Standard length - mean (range) cm	49	66.4 (59.0–73.5)	50	67.1 (61.0–75.0)	97	66.4 (59.0–69.5)	98^c^	66.2 (59.0–69.5)	63	73.3 (70.0–82.0)	63	72.8 (70.0–78.0)
Body weight - mean (range) kg	49	8.6 (5.6–11.2)	50	8.8 (5.8–11.6)	97	8.7 (5.6–12.8)	99	8.8 (5.6–11.7)	63	11.5 (7.4–17.0)	63	11.6 (9.0–14.6)
Body mass index - mean (range)	49	28.0 (21.4–34.4)	50	27.9 (20.6–36.8)	97	28.4 (21.4–36.8)	98^c^	29.0 (20.6–38.4)	63	28.3 (20.4–41.9)	63	29.2 (24.3–35.5)
Parasitic burden												
Hookworm prevalence - count (%)	49	0.0 (0.0%)	50	0.0 (0.0%)	97	53 (54.6%)	96^c^	51 (53.1%)	63	57 (90.5%)	63	54 (85.7%)
Lice prevalence - count (%)	49	13 (26.5%)	50	14 (28.0%)	97	34 (35.1%)	99	35 (35.4%)	63	31 (49.2%)	63	33 (52.4%)
Lice intensity score - median (range)^a^^,^^d^	49	0 (0–3)	50	0 (0–3)	97	0 (0–4)	99	0 (0–3)	63	0 (0–6)	63	1 (0–6)

Among the prepatent pups <14d old (negative fecal hookworm smears), 86 were part of the <70 cm standard length cohort and 13 were part of the ≥70 cm cohort. All within age cohort comparisons were deemed non-significant at the 5% level.

a Strong evidence of the continuous variable not being normally distributed – treatment group equalities were compared using non-parametric statistical test (Wilcoxon rank-sum test).

b Predicted age used where birth date was missing.

c Variation from stated n-values due to absence of parameter assessment at recruitment: standard length (and body condition index) was not determined in one treated pup <70 cm in season 2019; patent hookworm infection was not determined in three treated pups <70 cm (one in season 2019 and two in season 2020–1).

d Lice intensity score is a subjective assessment of lice infestation intensity, ranging from 0 (no lice) to a maximum of 9.

**Table 2:** Recruitment median values (and ranges) of hematological parameters for treatment groups of Australian sea lion pups for each age cohort.

	Prepatent pups < 14 d old (*n* = 99)				Pups < 70 cm standard length (*n* = 196)				Pups ≥ 70 cm standard length (*n* = 126)			
Variable [mean (range)]	Control		Treatment		Control		Treatment		Control		Treatment	
	*n*	Estimate	*n*	Estimate	*n*	Estimate	*n*	Estimate	*n*	Estimate	*n*	Estimate
PCV (L/L)	43	0.400 (0.270–0.520)	45	0.400 (0.310–0.520)	89	0.360 (0.210–0.520)	92	0.360 (0.210–0.520)	60	0.345 (0.200–0.440)	58	0.340 (0.190–0.460)
RBC (x10^12^/L)	83	3.84 (1.64–5.00)	89	3.74 (1.98–5.52)	83	3.84 (1.64–5.00)	89	3.74 (1.98–5.52)	57	3.70 (2.80–4.78)	55	3.64 (2.94–4.60)
Hemoglobin (g/L)	83	126.0 (70.0–172.0)	89	128.0 (68.0–192.0)	83	126.0 (70.0–172.0)	89	128.0 (68.0–192.0)	57	122.0 (98.0–166.0)	55	118.0 (78.0–152.0)
Reticulocytes (10^9^/L)	79	35.6 (3.9–269.4)	88	28.3 (0.0–234.7)	79	35.6 (3.9–269.4)	88	28.3 (0.0–234.7)	55	62.4 (3.9–218.4)	54	70.3 (17.2–182.7)
nRBC (10^6^/L)	81	55.0 (0.0–1280.9)	86	50.8 (0.0–1212.1)	81	55.0 (0.0–1280.9)	86	50.8 (0.0–1212.1)	57	89.5 (0.0–891.5)	52	136.3 (0.0–1544.0)
Platelets (10^9^/L)	80	351.0 (46.0–854.0)	86	346.0 (80.0–896.0)	80	351.0 (46.0–854.0)	86	346.0 (80.0–896.0)	57	416.0 (116.0–828.0)	54	446.0 (68.0–702.0)
WBC (10^9^/L)	82	11.84 (4.58–25.26)	88	12.16 (5.27–25.00)	82	11.84 (4.58–25.26)	88	12.16 (5.27–25.00)	56	11.71 (5.41–24.89)	54	11.34 (6.93–21.26)
Neutrophils (10^9^/L)	82	6.35 (1.70–20.00)	88	7.10 (1.00–18.7)	82	6.35 (1.70–20.00)	88	7.10 (1.00–18.7)	57	5.90 (1.30–18.3)	54	5.75 (1.80–12.2)
Lymphocytes (10^9^/L)	83	3.20 (1.60–6.40)	89	3.20 (0.70–7.10)	83	3.20 (1.60–6.40)	89	3.20 (0.70–7.10)	57	3.70 (1.40–7.1)	54	3.45 (1.40–6.90)
Monocytes (10^9^/L)	82	0.60 (0.00–1.90)	89	0.60 (0.10–1.90)	82	0.60 (0.00–1.90)	89	0.60 (0.10–1.90)	57	0.60 (0.10–1.70)	53	0.60 (0.10–1.40)
Eosinophils (10^9^/L)	82	0.80 (0.00–4.40)	88	0.80 (0.0–4.20)	82	0.80 (0.00–4.40)	88	0.80 (0.0–4.20)	56	1.3 (0.20–4.80)	52	1.45 (0.40–3.40)
Total plasma protein (g/L)	89	61.0 (42.0–83.0)	91	60.9 (40.0–82.0)	89	60.0 (42.0–83.0)	91	60.0 (40.0–82.0)	60	62.0 (46.0–92.0)	58	63.0 (48.0–92.0)

Among the prepatent pups <14d old (negative fecal hookworm smears), 86 were part of the <70 cm standard length cohort and 13 were part of the ≥70 cm cohort. All within age cohort comparisons were deemed non-significant at the 5% level. There was strong evidence for all variables being non-normally distributed. Median value and range are therefore reported, and treatment group equalities were compared using non-parametric statistical test (Wilcoxon rank-sum test). PCV: packed cell volume; RBC: total red blood cell count; WBC: total white blood cell count; nRBC: nucleated cell blood cell count.

### 3.2. Treatment effect on estimated hookworm prevalence and incidence of clearance

Patent hookworm infection was not detected in any treated pup at any live recapture. Regardless of pup age cohort, topical ivermectin significantly reduced hookworm prevalence at first recapture by at least 98%, the estimated relative risk reduction (*p* ≤ 0.001; Table 3). No evidence of interaction between treatment effectiveness (based on estimated hookworm prevalence) and season for any age cohort could be found. However, season 2020–1 had significantly higher predicted hookworm prevalence at first recapture than season 2019 in the <14 d (51.1% [46.7–55.5%] versus 35.1% [23.7–46.4%]; *p* = 0.043) and <70 cm (46.4% [42.7–50.1%] versus 35.6% [29.2–42.0%]; *p* = 0.015) age cohorts. These findings coincided with younger median pup age [and range] at first recapture for season 2020–1 versus 2019 in the <14 d (39 d [25–95 d] versus 47 d [33–71 d]) and <70 cm (46 d [25–103 d] versus 50 d [33–111 d]) cohorts. For the <70 cm cohort, there was a significant interaction between treatment effectiveness and age at first recapture, whereby a significant treatment benefit was no longer supported by 80 d of age at first recapture (coinciding with the natural elimination of hookworm in control pups). In pups with patent hookworm infection at recruitment, treatment also significantly increased hookworm clearance before first recapture by at least 98% (*p* < 0.001; Table 3). As expected, given natural hookworm clearance at 2–3 months of age (20), older age at first recapture was marginally associated with increased hookworm clearance for the two older <70 cm (OR 1.13 [1.00–1.28]; *p* = 0.047), and ≥70 cm (OR 1.05 [1.00–1.11]; *p* = 0.042) age cohorts.

**Table 3:** Adjusted effectiveness of topical ivermectin treatment in Australian sea lion pups for each age cohort using either the hookworm prevalence at first recapture or incidence of hookworm clearance during the recruitment-to-first recapture period.

	Hookworm prevalence or clearance estimate (95%CI)^e^					Effect estimate (95%CI)	
Recruitment age cohort	*n*	Control	*n*	Treatment	*p*-value	Odds ratio	Treatment effect ^a^
Hookworm prevalence at first recapture							
Prepatent pups <14d old^b^	43	85.5% (76.0–94.9%)	41	1.4% (0.0–5.2%)	<0.001	0.002 (0.000–0.003)	+98.4% (94.5–100.0%)
Pups <70 cm standard length	83	84.5%^c^ (78.0–91.1%)	86	1.2%^c^ (0.0–3.5%)	0.001	0.002 (0.000–0.004)	+98.6% (96.1–100.0%)
Pups ≥70 cm standard length	59	81.3% (72.3–90.3%)	61	0.9% (0.0–3.4%)	<0.001	0.002 (0.000–0.004)	+98.9% (96.3–100.0%)
Incidence of hookworm clearance during the recruitment-to-first recapture period^d^							
Pups <70 cm standard length	45	18.4% (9.5–27.2%)	47	99.1% (96.7–100.0%)	<0.001	0.002 (0.000–0.004)	+98.9% (96.4–100.0%)
Pups ≥70 cm standard length	53	19.6% (9.8–29.3%)	52	98.9% (96.1–100.0%)	<0.001	0.003 (0.000–0.004)	+98.7% (95.7–100.0%)

Prepatent pups (<14d old and negative fecal hookworm smears at recruitment) were part of the other two cohorts defined by standard length alone. Age at recruitment (median [range]) for the <14d prepatent, <70 cm standard length and ≥70 cm standard length pup cohorts were 10d [4–13d], 14d [4–33d], and 24d [8–63d], respectively. Recruitment-to-first recapture period length (median [range]) for the same three age cohorts were 32d [16–86d], 31d [15–94d], and 31d [16–111d], respectively.

a The adjusted effect estimate was calculated using the relative risk reduction associated with treatment versus control.

b As these pups were prepatent at recruitment, the prevalence at first capture corresponds to the incidence of infection until first recapture.

c The final model includes a significant interaction between treatment and pup age. The reported estimates correspond to the prevalence of an average aged pup in either treatment group.

d Treatment effectiveness for the incidence of clearance (recruitment-to-first recapture period) calculation in pups with patent infection at recruitment used the predicted proportion of patent infection at recapture (the complement of clearance from recruitment-to-first recapture).

e Hookworm prevalence and incidence estimates were calculated using Firth’s variant of the multifactorial logistic regression. The base additive model included treatment group, season, and pup age at first recapture. Ancillary factors assessed for significance were sex; bodyweight, standard length, and body mass index at first recapture; first recapture fieldtrip; and the recruitment-to-first recapture period length.

### 3.3. Treatment effect on growth

**Figure 3:**
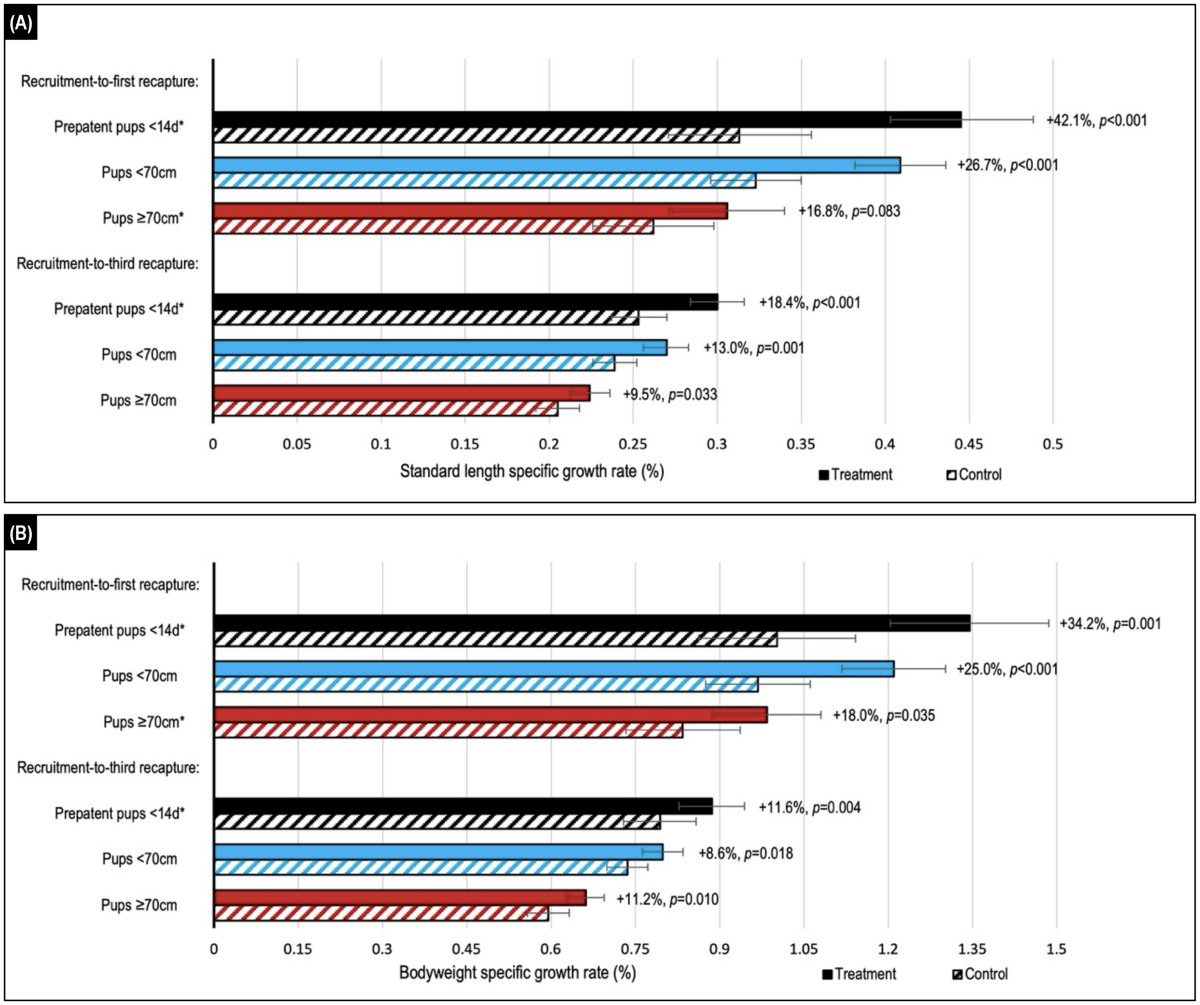
Comparison of estimated median values for **(A)** standard length and **(B)** bodyweight daily specific growth rates (SGRs) in Australian sea lion pups after either topical ivermectin application and early hookworm elimination (treated), or no treatment (control). Treatment effect (calculated as the relative difference in daily SGR between treatment and control groups) and *p*-value are shown to the right of the treatment bars. Stratification by age showed the highest relative benefits in the youngest <14d age cohort of prepatent pups. Assessment across the recruitment-to-first recapture (median 31 d, range: 15–111 d) and recruitment-to-third recapture (median 95 d, range: 76–195 d) periods showed the greatest benefits in the month immediately after treatment and persistence of benefits to 3  months post-treatment for all age cohorts. Daily SGRs were calculated as the natural log of each morphometric at the start of the period, minus that at the end of the period, divided by period length in days. Error bars indicate the estimated 95% confidence intervals. *A significant interaction between treatment and follow-up period length in days was apparent, therefore estimates are reported for the daily SGR at the median length of the follow-up period.

During the recruitment-to-first recapture period, topical ivermectin treatment resulted in higher growth rates based on changes in standard length and bodyweight, reaching significance (*p* ≤ 0.035) for all age cohort comparisons, excepting standard length in the ≥70 cm cohort (*p* ≤ 0.083; Figure 3; Supplementary Table S1). The youngest <14 d cohort benefited the most from treatment, with a relative increase in bodyweight and standard length growth of approximately 35–40% compared to the <70 cm (approximately 25%) and ≥70 cm (approximately 18%) cohorts for the same period. The treatment benefit decreased after the first recapture, and while mostly trending positive it was usually not significant in the subsequent follow-up periods. An approximately 10–15% overall beneficial impact of treatment persisted for the entire follow-up period (recruitment-to-third recapture) for all age cohorts, with the highest relative benefit seen in the youngest <14 d cohort. There was no significant interaction of season with treatment when assessing growth (*p* > 0.05).

### 3.4. Treatment effect on hematological parameters

#### 3.4.1. Red blood cell and platelet counts

**Figure 4:**
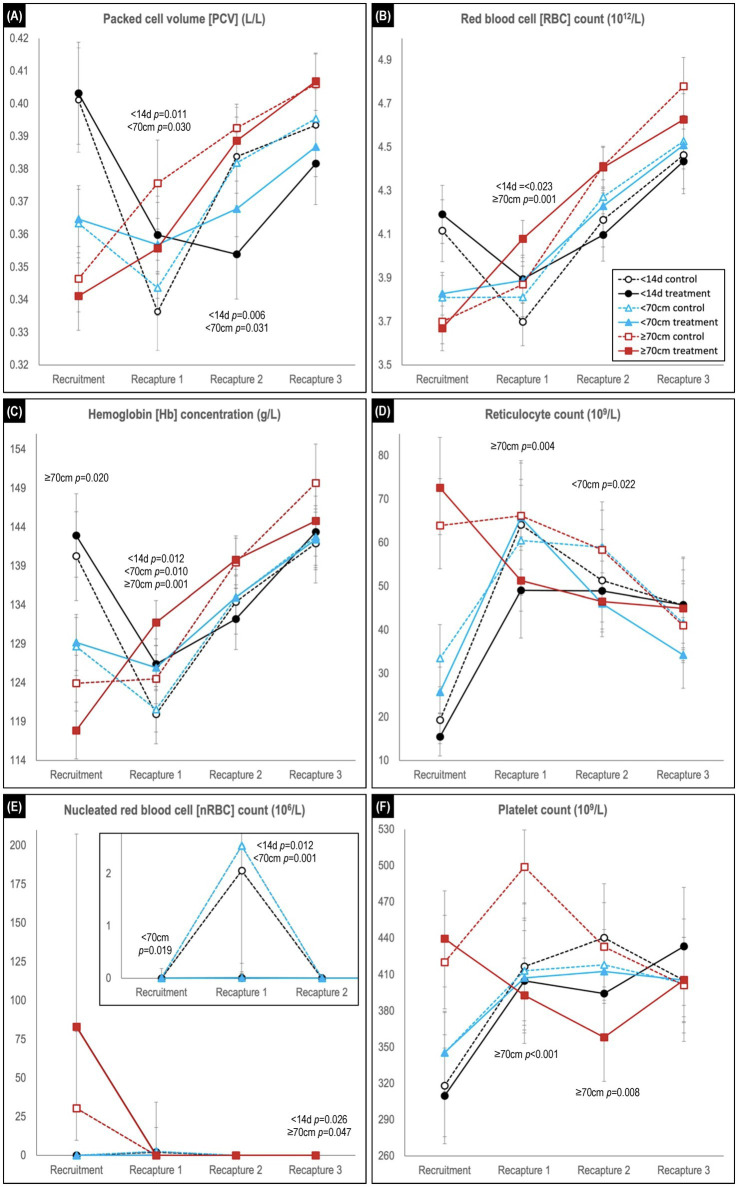
Comparison of predicted mean or median* values (and 95% confidence intervals) for **(A)** packed cell volume, **(B)** red blood cell count, **(C)** hemoglobin concentration, **(D)** reticulocyte count, **(E)** nucleated red blood cell count, and **(F)** platelet count in hookworm treated and control groups of Australian sea lion pups. Results are stratified by pup recruitment age (size) cohorts – <14 d prepatent, <70  cm standard length and ≥70  cm standard length. Significant between treatment group differences within any of the three age cohorts are listed adjacent to the respective capture. Differences between treatment groups within the same age cohort quantify the impact of hookworm infection, which is superimposed on temporal changes attributable to age-related hematological ontogenesis in maturing pups. The median interval (and range) for the recruitment-to-recapture 1, recapture 1-to-recapture 2, and recapture 2-to-recapture 3 periods for the combined cohorts were 31 d (15–111 d), 31 d (20–99 d), and 33d (20–99 d), respectively. **E** shows a magnification of the results for the <14 d and <70 cm cohorts for the first 3 captures. *Transformed model estimates and their associated 95% CI boundaries were back transformed prior to reporting and should be interpreted as medians rather than means (refer to Supplementary Table S2 for specific detail values of transformed models).

**Figure 5:**
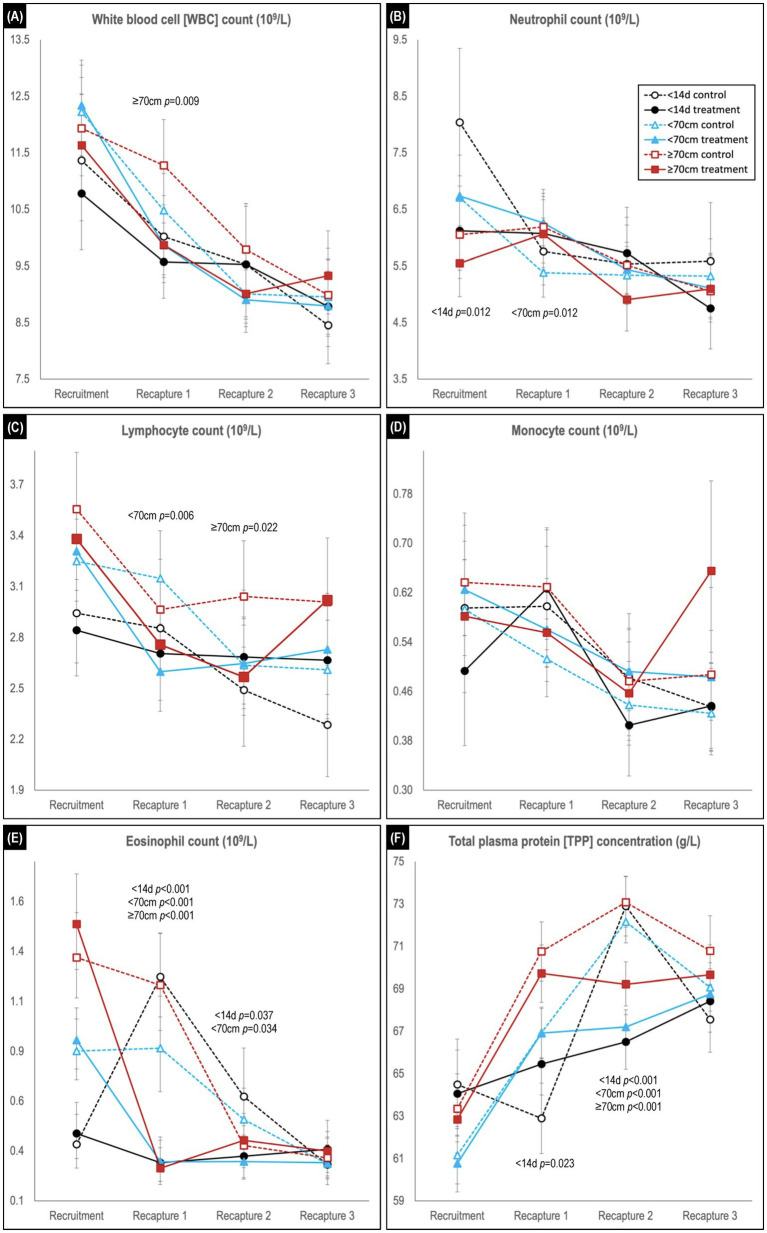
Comparison of predicted mean or median* values (and 95% confidence intervals) for **(A)** white blood cell, **(B)** neutrophil, **(C)** lymphocyte, **(D)** monocyte, and **(E)** eosinophil counts, and for **(F)** total plasma protein concentration in hookworm treated and control groups of Australian sea lion pups. Results are stratified by pup recruitment age (size) cohorts – <14 d prepatent, <70 cm standard length, and ≥70  cm standard length. Significant between treatment group differences within any of the three age cohorts are listed adjacent to the respective capture. Differences between treatment groups within the same age cohort quantify the impact of hookworm infection, which is superimposed on temporal changes attributable to hematological ontogenesis in maturing pups. The median interval (and range) for the recruitment-to-recapture 1, recapture 1-to-recapture 2, and recapture 2-to-recapture 3 periods for the combined cohorts were 31 d (15–111d), 31 d (20–99 d), and 33 d (20–99 d), respectively. *Transformed model estimates and their associated 95% CI boundaries were back transformed prior to reporting and should be interpreted as medians rather than means (refer to Supplementary Table S2 for specific detail values of transformed models).

Comparison of temporal changes in predicted mean (or median) values of hematological parameters likely to be impacted by hookworm disease is shown in Figures 4, 5 (refer to Supplementary Table S2 for individual parameter values). The parameters indicative of red blood cell number, and therefore anemia (PCV, RBC count and Hb concentration; Figures 4A–C), showed similar trends, exemplified by the PCV. At recruitment, PCV was highest for the <14 d cohort and lowest for the ≥70 cm cohort. In the <14 d and <70 cm cohorts, levels declined for both treatment groups in the recruitment-to-first recapture period, but to a significantly greater degree for untreated pups (<14 d cohort *p* = 0.011; <70 cm cohort *p* = 0.030), followed by increases in the subsequent two periods for these age cohorts (as occurred across all follow-up periods in the ≥70 cm cohort). For the <14 d cohort, the relative decline in PCV between recruitment and first recapture in untreated pups was approximately 50% greater than in the treated pups (16.1% vs. 10.8%, respectively). Likewise, untreated pups showed 43% and 25% greater declines in RBC count (10.1% versus 7.1%) and Hb concentration (14.5% and 11.6%), respectively. In the first-to-second capture period, the increase in PCV of the control group exceeded that of the treated group, such that significantly higher values (*p* = 0.006 and *p* = 0.031, respectively) were apparent for the <14 d and <70 cm cohorts at the end of the period. A similar non-significant trend for higher PCV in the control group of the oldest ≥70 cm cohort was also apparent in the recruitment-to-first recapture and first-to-second recapture periods. By trial conclusion, indicators of red cell number were frequently highest in the ≥70 cm cohort and lowest in the <14 d cohort. The greater rebound in red cell number in control groups suggested by PCV was less apparent in Hb concentration and RBC count.

Reticulocyte counts at recruitment were lowest for the <14 d cohort and highest for the ≥70 cm cohort (Figure 4D). In the recruitment-to-first recapture period, counts subsequently increased for the <14 d and <70 cm cohorts and remained stable or decreased for the ≥70 cm cohorts, reaching similar levels across all age cohorts by study conclusion. While counts at first and second recapture were most commonly lower in treated groups, significant between group differences were limited to lower counts in the treated group at first recapture for the ≥70 cm cohort (*p* = 0.004) and at second recapture for the <70 cm cohort (*p* = 0.022). For nRBC count (Figure 4E), counts at first recapture were significantly higher in control versus treated groups for the <14 d (*p* = 0.012) and <70 cm (*p* = 0.001) cohorts (noting the latter cohort’s absence of random allocation at recruitment when using multifactorial modelling rather than simple direct between group comparison). This difference was not apparent for both control and treated pups at recruitment in the ≥70 cm cohort.

At recruitment, platelet count was lowest in the <14 d cohort and highest in the ≥70 cm cohort (Figure 4F). For the two youngest cohorts (<14 d and <70 cm), increasing counts in the treated groups in the recruitment-to-first recapture period paralleled those in the control group, before plateauing in the first-to-second recapture period – a change less apparent in the respective control groups. In the ≥70 cm cohort by contrast, lower platelet counts in the treated pups were significant at the first (*p* < 0.001) and second (*p* = 0.008) recaptures, prior to approximating those of the control group (and other cohorts) by the study conclusion.

#### 3.4.2. White blood cell counts

Across the study, a trend for decreasing WBC count was observed in all age cohorts, characterized usually by declines in each of the individual leukocytes (Figures 5A–E). Within each age cohort, counts tended to be lower in the treated groups, although significance was apparent only for the ≥70 cm cohort at first recapture (*p* = 0.009). For the individual leukocytes at recruitment, neutrophil counts were generally highest, and lymphocyte and eosinophil count lowest in the youngest <14 d age cohort, while monocyte counts did not show a clear delineation. For neutrophil, lymphocyte, and monocyte counts, significant between treatment group differences at recaptures were limited to higher neutrophil count in treated pups of the <70 cm cohort at first recapture (*p* = 0.0012), and lower lymphocyte count in treated pups for the <70 cm cohort at first recapture (*p* = 0.006) and ≥ 70 cm cohort at second recapture (*p* = 0.022). In contrast, in the recruitment-to-first recapture period across all age cohorts, eosinophil counts in treated pups decreased significantly (*p* < 0.001) compared with control pups – immediately declining to a level comparable with the eventual control group baseline (Figure 5E). These decreases contrasted with the changes observed in the control groups – a rapid increase in the <14 d cohort, a plateau in the <70 cm cohort and a lesser decline in the ≥70 cm cohort. At second recapture, eosinophil levels remained significantly higher in the control groups of the <14 d (*p* = 0.037) and <70 cm (*p* = 0.034) age cohorts, before declining to comparable levels with the treated animals at third recapture.

#### 3.4.3. Total plasma protein concentration

In the recruitment-to-first recapture period, the treated group TPP concentration increased in parallel with that of the control group for the <70 and ≥70 cm cohorts, but not for the <14 d cohort [in which the control group showed a significant initial decline in TPP (*p* = 0.023; Figure 5F)]. In the subsequent second-to-third recapture period, a similar (delayed) increase for the younger <14 d cohort’s control group was not paralleled by its treated group, and levels also plateaued in the treated groups of the other two age cohorts. Consequently, at second recapture TPP concentration was similar across all three age cohort control groups, and significantly higher than for the respective treated groups (*p* < 0.001 for all age cohorts). By study conclusion there were no significant differences between treated and control groups for TPP concentration in any age cohort.

Significant interactions between season and treatment were only seen in a small number of comparisons (5.6%; *n* = 144), half of which were at recruitment – WBC, neutrophil and monocyte counts in the <14 d cohort and nRBC count at recruitment in the <70 cm cohort. The other four significant interactions were at recapture (3.7%; *n* = 108; post-treatment) – PCV and nRBC count at second recapture for the <14 d cohort; reticulocyte count at second recapture for the <70 cm cohort; and monocyte count at third recapture for the ≥70 cm cohort.

## 4. Discussion

Hookworm infection is an impactful endemic disease of Australian sea lion pups – occurring in all pups, contributing to substantial morbidity (22), and having a significant positive correlation with colony pup mortality based on infection intensity (20). By addressing the logistical limitations of previous hookworm treatment trials in the Australian sea lion, this study suggests a growth benefit following early hookworm elimination and better defines the hematological changes attributable to hookworm infection. Growth benefits were greatest in the month immediately post-treatment, persisted for at least 3 months, and were optimal for pups treated prior to patent infection (11–14 d of age). Similarly, early hookworm elimination resulted in immediate hematological benefits with reduced systemic eosinophilic inflammation and elimination of the decline in RBC count associated with patent infection. Importantly, the benefits of hookworm elimination on growth and health parameters were independent of season.

### 4.1. Treatment effect on hookworm prevalence and incidence

The estimated effectiveness of topical ivermectin approaching 100% for elimination of hookworm infection in Australian sea lion pups further supports findings of a prior trial of this formulation (21). Treatment was effective as a single application in pups at any age – including those with prepatent disease – and was well tolerated, with no death attributed to its application. The effectiveness of topical ivermectin parallels that of the injected formulation, evaluated in earlier Australian sea lion studies (21, 24) and in studies in other pinniped species (25, 26, 32–34). The present study also demonstrated topical ivermectin’s benefits across lower and higher mortality breeding seasons. Seasonal differences in hookworm prevalence (significantly higher in season 2020–1) and incidence of clearance (trending lower in season 2020–1) are explained by the recruitment of a younger cohort in season 2020–1 – more untreated animals still of patent infection age (approximately 11–60 days) at first recapture. Similarly, the natural clearance of hookworm infection in untreated pups from approximately 60–90 days explains the progressive decline of treatment effectiveness with increasing pup age at the post treatment assessment (first recapture).

### 4.2. Treatment effect on growth

In contrast to previous studies in the Australian sea lion, this larger scale study provided evidence of a significant treatment-related growth benefit that was both immediate and persisted for at least 3 months. Demonstration of a growth benefit following early hookworm elimination in pups is consistent with observations in other seal species, including those in the northern fur seal (*Callorhinus ursinus*) (26), New Zealand sea lion (*Phocarctos hookeri*) (25), and South American fur seal (35). In the earlier treatment trials in Australian sea lion pups, a significant growth benefit was either not apparent (24) or only observed for a short term (15–24 d post-treatment) (21). In a more recent study of New Zealand sea lions, trends for treatment-related improvement in weight and length were also detected, although these differences were not significant (34). Differences between the present study and others may reflect differences in sample size, methodology for assessing growth, species-specific host and parasite attributes, and age of treatment and recapture (21). Multifactorial regression of daily SGR (for two parameters of growth) was favored in the present study to account for the potential effect of pup age at treatment, including baseline morphometrics, as well as follow-up period length.

Key growth-related outcomes unique to this study highlighted optimization of treatment benefit by treating pups prior to infection patency. Notably, even a 4 days increase in median recruitment age and inclusion of pups with patent disease (comparing the <70 cm and <14 d cohorts) decreased treatment benefit due to limiting juvenile (L4 stage larvae) parasite-associated damage to the intestines and subsequent nutrient deprivation, and preventing any disease caused by adult parasites. In comparison, pups in the oldest age cohort (median recruitment age 24 days) had experienced a median of approximately 2 weeks of patent disease prior to treatment. Importantly, this study demonstrated persistence of significant growth benefits beyond the age (approximately 60 days) (20) that untreated pups begin to naturally eliminate hookworm infection – again, even for the oldest age cohort. This suggests the potential for persistence of a treatment-related growth benefit up to weaning (18 months of age), and possibly into the juvenile and adult life stages. Such an outcome could have substantial implications for longer term health, reproductive fitness, and survival – key objectives of infection control in Australian sea lion pups. Multiple studies have confirmed a significant positive correlation between pup size (weight, body condition) at weaning and post-weaning survival, including in grey (*Halichoerus grypus Fabricius 1791*) (36, 37) and harbour (*Phoca vitulina*) (38) seals to one year of age, and Hawaiian monk (*Monachus schauinslandi*) (39) and Northern fur (*Callorhinus ursinus*) (40) seals to 2 years of age. Additionally, only one treatment comparison in this study demonstrated a higher growth rate in the untreated group (bodyweight in the second recapture-to-third recapture period for the <14 d cohort; *p* = 0.029), providing little support for compensatory growth (41) in untreated pups following natural hookworm elimination during the 3 month follow-up period. Using the commencement of hookworm elimination from 60 d of age, the median (range) length of the postpatent period monitored in the present study was up to 44 d (26–143 d), 53 d (26–143 d), and 62 d (28–136 d) for the <14 d, <70 cm, and ≥70 cm age cohorts, respectively – presumed sufficient for detection of compensatory growth given a significant treatment-related benefit from early hookworm elimination was apparent within a month. Ongoing monitoring of growth rates in Australian sea lions becomes logistically challenging with increasing age. However, the philopatric nature of the species means that persistent treatment-related growth and health benefits manifesting as increased longer-term survival could be assessed at the closely monitored Seal Bay colony.

### 4.3. Treatment effect on hematological parameters

Previous studies have developed hematological reference values for Australian sea lion pups with patent and post-patent hookworm infection (22), and defined the impact of patent infection by comparison with disease-free (anthelmintic treated) individuals (21). Based on these prior studies, regenerative anemia, hypoproteinemia or hyperproteinemia, and lymphocytic and/or eosinophilic inflammation have been associated with patent hookworm infection. In the present study, the extended 3 months follow-up period, stratification into three recruitment (and therefore treatment) age cohorts, inclusion of infection-free treated and control groups, and multifactorial modelling to account for the impact of non-treatment factors, all served to further explore the pathological impact of hookworm infection from physiological changes attributable to hematological ontogenesis.

#### 4.3.1. Red blood cell and platelet counts

Detection of higher RBC count in younger pups, followed by an early post-natal decline, is consistent with previous observations in the Australian sea lion and other otariid species (22, 42, 43). Similar change is apparent in terrestrial mammals and attributed to a physiological decline associated with the rapid transition from a low (placental-based) to high (lung-based) oxygen environment at birth (44, 45). Mechanisms include decreased RBC production following the negative feedback from higher tissue oxygen saturation on erythropoietin (RBC stimulatory factor) production, a shortened lifespan of fetal and neonatal RBCs versus adult RBCs, and the dilutionary effect of expanded blood volume associated with rapid perinatal growth [reviewed in (46, 47)]. The present study demonstrates an additional significant decline in RBC count in untreated versus treated pups (25–50%, depending on RBC parameter), quantifying the pathological contribution directly attributable to hookworm infection. Subsequent increases in RBC count in treated pups suggests the restoration of erythropoietin stimulation of RBC production in response to the physiological decline in hemoglobin levels (46). Untreated pups demonstrate a significantly greater (second recapture PCV: *p* = 0006 and *p* = 0.001 for <14 d and <70 cm cohorts, respectively) regenerative response from a comparatively more severe anemia resulting from the additional pathological contribution of hookworm infection. Higher reticulocyte and nRBC counts in the untreated pups at the time of the RBC nadir support an increased level of erythropoietin-driven RBC production. It remains to be determined if this rebound increase in RBC production is consequential to host–parasite co-evolution and clinically advantageous to pups – for instance, providing a survival benefit during this period. Given that pup mortality occurs mostly in the first month of life (19), which coincides with the initial significant RBC decline apparent in hookworm-infected pups, it is more likely that improvement in pup health resulting from early hookworm elimination will translate to improved pup survival.

Highest platelet counts at recruitment apparent in the older versus younger pups, and a treatment-independent increase in the first month post-recruitment in the younger pups, suggest initial platelet increases are driven by ontogenesis. The subsequent stabilization of platelet counts in younger treated pups to levels approximating those at the study conclusion, differed from further mild increases in the control group. Additionally, treated pups in the oldest ≥70 cm cohort showed significantly lower platelet counts compared with untreated pups for two months post-treatment. Both observations suggest a pathological contribution of hookworm to a relative platelet increase (thrombocytosis) during the latter patent-and early post-patent period. Potential contributors to a thrombopoietin-driven reactive thrombocytosis include (1) a direct response to ongoing platelet utilization related to hookworm feeding and intestinal hemorrhage, and (2) an indirect response driven by other inflammatory cytokines released in response to hookworm-associated intestinal inflammation. The latter is supported by a temporal relationship with higher eosinophil and lymphocyte counts in the untreated pups at first and second recapture. These results are consistent with the role of platelets in chronic intestinal mucosal inflammation and coagulation [reviewed in (48)]. Further, they add to the outcomes of an earlier study in the Australian sea lion, in which platelets trended lower in treated versus control pups at 15–24 d post treatment and were similar by 36–41 d (21).

#### 4.3.2. White blood cell counts

Consistent with findings in the Steller sea lion (*Eumetopias jubatus*) in the first month of life (49) and contrasting those in the South American fur seals in the first 2 months of life (42), WBC count in the Australian sea lion pups decreased in the first 3–4 months of life, independent of hookworm infection status. Decreasing counts attributable to ontogeny were typically characterized by a decrease in each of the individual WBCs – apart from the marked increase in eosinophil count attributed to the onset, and persisting for the duration of, patent hookworm infection. The close temporal relationship between patent hookworm infection and systemic eosinophilic inflammation, is previously reported in treatment trials in otariid species (21, 24, 50). Treatment-related changes in the remaining WBCs were less significant, excepting the frequently higher lymphocyte counts in the untreated pups, also reported in those earlier studies. Significant between group differences in lymphocyte count coincided with mid-to late patent hookworm infection, indicating antigenic stimulation and a host immune response. This has been confirmed in an immunological study of hookworm infected Australian sea lions, showing a positive association of increasing pup age with B-lymphocyte count and immunoglobulin G (IgG; antibody) concentration across the period of hookworm patency (51). While there is the potential for early hookworm elimination to negatively impact lymphocyte ontogenesis, comparable lymphocyte counts in treated and control pups of the oldest two age cohorts by the study conclusion suggest any impact is likely to be short term.

#### 4.3.3. Total plasma protein concentration

Results of this study, particularly for TPP concentration, highlight the importance of including age and other contributing factors in modelling of disease or treatment impact on pup hematological parameters – an outcome also evident in a recent disease investigation in the New Zealand sea lion (50). The declining TPP concentration in the recruitment-to-first recapture period in untreated pups of the youngest <14 d cohort contrasts with a mild increase in treated pups of that cohort, and more rapid increases in treated and untreated pups of the older two age cohorts. Treatment-independent increases in the older cohorts favor a physiological (ontogeny-related) increase, with a significant superimposed impact from hookworm infection in the youngest cohort (median [range] age at first recapture of 42 d [25–95 d] – mid-patency; time post-recruitment 32 d [16–86 d]). Lower TPP levels have previously been reported in Australian sea lion pups with patent, versus pre-or postpatent hookworm infection, and reasonably attributed to intestinal hemorrhage (in association with lower RBC counts) caused by the blood-sucking nature of the intestinal parasite (22). Lower TPP has also been reported with hookworm-infected (versus treated) New Zealand sea lion pups, albeit with an important age-related influence (50). In contrast, the two previous treatment trials in the Australian sea lion showed either significantly higher TPP concentrations in the untreated pups at 15–24 d (but not at 36–41 d) post-treatment (21), or a non-significant trend for the same outcome at 27–67 d post-treatment (24). These outcomes were suggested as reflective of positive acute phase inflammatory responses or the influence of unrelated factors (e.g., lice infestation). In the present study, a longer follow-up period identified significantly higher TPP concentrations (*p* < 0.001) in untreated pups of all three age cohorts at second recapture (combined median time [range] post recruitment 63 d [41–147 d]; ages 72 d [51–128 d], 82 d [51–142], and 88 d [55–168 d] for the <14 d, <70 cm, and ≥70 cm cohorts, respectively – all approximating the age of natural hookworm elimination). The above mentioned study of the immune response to hookworm infection in the Australian sea lion provides some explanation for the apparent discrepancies and suggests an inflammatory contribution to increased TPP concentration in untreated pups (51). In that study, the start of the patent period was marked by a peak in IL-6 (a pro-inflammatory cytokine) gene expression indicating early stage inflammation, followed initially by an increase in positive acute phase proteins (proteins increasing early in the inflammatory process), and then by the increase in B-lymphocyte count and IgG production. Observations were consistent with an antihelminth inflammatory response, peaking at the time of natural hookworm elimination. Superimposed on this inflammatory contribution to TPP concentration was an age-related increase in albumin concentration, reaching a maximum in the postpatent period (51). This followed an initial decline in the very early stages of the inflammatory response (confirming albumin as a negative acute phase protein in the Australian sea lion). In the present study, therefore, is the potential for (1) the initial reduction in the TPP concentration in the youngest <14 d prepatent cohort to reflect intestinal protein loss and a negative acute phase response to the commencement of patent disease, and (2) the comparative plateauing observed in treatment groups at the time of natural hookworm elimination in untreated animals to indicate amelioration of the hookworm association inflammatory response. A reduction in TPP concentrations in untreated groups of all cohorts to levels comparable with those in the treated groups indicates an end to the hookworm-associated inflammatory response by the study conclusion – consistent with observations in the eosinophil count.

The few hematological parameters of health where a seasonal interaction with treatment outcome was detected statistically, were inconsistent across cohorts and not considered clinically relevant. Consequently, independent of whether treatment was administered in a lower or higher mortality season, early hookworm elimination provided immediate improvements in hematological analytes frequently impacted by hookworm disease. Ongoing monitoring of these individuals will indicate whether these improvements in health will translate into improved pup survival. The absence of significant differences in the hematological parameters of treated and untreated pups by study conclusion (nRBC count the exception, likely impacted by factors unrelated to hookworm disease) highlights the absence of any positive or negative longer term hematological alterations consequential to anthelmintic treatment in this species.

### 4.4. Conclusion

This study indicates that optimal growth and health outcomes from early hookworm elimination in the Australian sea lion are attained by treatment of pups in the prepatent period (first 2 weeks of age) and achievable in both lower and higher mortality breeding seasons. Intervention in pups of this age was readily achievable using an easily applied topical anthelmintic and offers a critical opportunity to mitigate the negative health impacts of an endemic disease contributing to substantial morbidity and mortality in this species. At an individual level, hookworm elimination improved pup health and therefore animal welfare. At a population level, the goal of intervention is to contribute to sustaining or increasing population size – addressing the high pup mortality in this species by improving overall pup health. Measuring the impact of hookworm infection to pup mortality and improving pup survival following early hookworm elimination are therefore two important next steps in the study of the endangered Australian sea lion.

## Data availability statement

The raw data supporting the conclusions of this article will be made available by the authors, without undue reservation.

## Ethics statement

The animal study was reviewed and approved by the University of Sydney Animal Ethics Committee (Protocol Number 2017/1260), The University of Sydney, Australia.

## Author contributions

RG and SL contributed to conception and design of the study. MF, RG, and SL collected and processed samples. SL performed the blood film reviews. SL and RG organized the database. SL and CC performed the statistical analysis. SL wrote the first draft of the manuscript. All authors contributed to the article and approved the submitted version.

## Funding

This study was partly funded by The Hermon Slade Foundation (HSF 16-03), the FS Quiney and BR Richards Bequests (Sydney School of Veterinary Science, The University of Sydney: 2016), and the Department for Environment and Water, South Australia (DEW).

## Conflict of interest

The authors declare that the research was conducted in the absence of any commercial or financial relationships that could be construed as a potential conflict of interest.

## Publisher’s note

All claims expressed in this article are solely those of the authors and do not necessarily represent those of their affiliated organizations, or those of the publisher, the editors and the reviewers. Any product that may be evaluated in this article, or claim that may be made by its manufacturer, is not guaranteed or endorsed by the publisher.
