# Supplementary material for: Mitigating disease risk in an endangered pinniped: early hookworm elimination optimizes the growth and health of Australian sea lion pups
Source: Front Vet Sci. 2023 Apr 25;10:1161185. doi: 10.3389/fvets.2023.1161185 (PMC10168540; doi:10.3389/fvets.2023.1161185)
Supplement: Supplementary file 1 [file Data_Sheet_1.docx]

Supplementary Material

Mitigating disease risk in an endangered pinniped: early hookworm elimination optimizes the growth and health of Australian sea lion pups

**Scott A. Lindsay, Mariel Fulham, Charles G.B. Caraguel, and Rachael Gray***

*** Correspondence:** Rachael Gray: [rachael.gray@sydney.edu.au](mailto:rachael.gray@sydney.edu.au)

# SUPPLEMENTARY DATA S1 Estimation of pup age

To predict the age at recruitment for pups of unknown birth date, a multifactorial linear regression was built using other available parameters from pups of known age. Predicted age was therefore specific to the pup cohort (and site and seasons) in the present study, intentionally optimised for use only in the present study.

## Method

For model building, the base additive model included pup sex and season, and ancillary factors were standard length, bodyweight, presence of lice and lice score, presence of patent hookworm infection, subjective body condition score, and body mass index (BMI). For consistency with a previous study, BMI was calculated using a similar method for investigation as an independent variable in modelling of other parameters (Lindsay et al., 2021). Ancillary factors were only retained, independent of significance, where they contributed to maximising the predictive value of the regression. A Box-Cox analysis was performed to explore the potential need of model outcome transformation to suit the assumptions of model’s residuals normal distribution and homoscedasticity. Outliers at the extremes of recruitment age were assessed for negatively impacting the predictive value of the regression.

## Results

When predicting age at recruitment, bodyweight was highly colinear with standard length (r=0.833) and was favored in the model building. The best model for age prediction (*r^2^*=0.63) was:

$$\boldsymbol{Age}^{\boldsymbol{recruitment}}\boldsymbol{=}\boldsymbol{e}^{\begin{aligned} \mathbf{1.932+0.088*BW-0.011*BMI-0.029*}\boldsymbol{Male}\mathbf{-0.131*}\boldsymbol{Season}\boldsymbol{2020}\mathbf{+0.630*}\boldsymbol{hookworm}\mathbf{-} \\ \mathbf{[0.026-0.920]*}\boldsymbol{BCS}\mathbf{+[0.064-0.537]*licescore} \end{aligned}}$$

where *BW*=bodyweight, *BMI*=body mass index, *Male*=2 for males and 1 for females, *Season2020*=1 if recruitment was season 2020-1 and 0 if season 2019, hookworm=1 if positive and 0 if negative, *BCS*=body condition score, and *licescore*=lice score, all covariates being measured at time of recruitment. Six known-age pups were excluded from the model estimation – three young outliers reported to be <4d old at recruitment were realistically 2-3 days older (the interval between two colony observations for new births), and three were extreme outliers (visual assessment using scatterplot) for standard length and/or bodyweight when comparing either parameter with age (one of whom was also postpatent rather than prepatent and negatively impacting the model – both testing negative for hookworm). Age at recruitment was natural log transformed to comply with the model residuals assumptions.

# SUPPLEMENTARY TABLE S1 Comparison of predicted mean values (and 95% confidence intervals) of bodyweight and standard length daily specific growth rates (SGRs) for treatment and control groups across each age cohort of Australian sea lion pups

|  | **Predicted means (95% CI) for daily SGR (%)** | | | | | |
| --- | --- | --- | --- | --- | --- | --- |
| **Recruitment age cohort** [median pup age at end of period (range)] | **n** | **Control** | **n** | **Treatment** | **Effect^a^** | ***p*-value** |
| **Recruitment-to-first recapture** | | | | | | |
| Prepatent pups <14d [42d (25-95d)] | | | | | | |
| Bodyweight (%)^b^ | 43 | 1.002 (0.863-1.142) | 41 | 1.345 (1.204-1.486) | +34.2% | 0.001 |
| Standard length (%)^b^ | 43 | 0.313 (0.271-0.356) | 41 | 0.445 (0.403-0.488) | +42.1% | <0.001 |
| Pups <70cm [48d (25-111d)] | | | | | | |
| Bodyweight (%) | 84 | 0.968 (0.875-1.061) | 86 | 1.210 (1.118-1.302) | +25.0% | <0.001 |
| Standard length (%) | 83 | 0.323 (0.296-0.350) | 85 | 0.409 (0.382-0.436) | +26.7% | <0.001 |
| Pups ≥70cm [56d (26-132d)] | | | | | | |
| Bodyweight (%)^b^ | 59 | 0.834 (0.733-0.936) | 61 | 0.984 (0.888-1.080) | +18.0% | 0.035 |
| Standard length (%)^b^ | 58 | 0.262 (0.226-0.298) | 61 | 0.306 (0.272-0.340) | +16.8% | ns |
| ­­­­ | | | | | | |
| **First recapture-to-second recapture** | | | | | | |
| Prepatent pups <14d [72d (51-128d)] |  |  |  |  |  |  |
| Bodyweight (%) | 36 | 0.659 (0.555-0.763) | 38 | 0.778 (0.677-0.879) | +18.1% | ns |
| Standard length (%)^c^ | 36 | 0.158 (0.116-0.199) | 38 | 0.285 (0.245-0.324) | +80.3 | 0.011 |
| Pups <70cm [82d (51-142d)] | | | | | | |
| Bodyweight (%) | 71 | 0.683 (0.604-0.763) | 77 | 0.719 (0.642-0.795) | +5.2% | ns |
| Standard length (%) | 71 | 0.206 (0.179-0.232) | 77 | 0.234 (0.208-0.259) | +13.5% | ns |
| Pups ≥70cm [88d (55-168d)] | | | | | | |
| Bodyweight (%) | 51 | 0.488 (0.398-0.577) | 55 | 0.592 (0.506-677) | +21.3% | ns |
| Standard length (%) | 50 | 0.169 (0.137-0.200) | 55 | 0.195 (0.166-0.225) | +16.0 | ns |
|  | | | | | | |
| **Second recapture-to-third recapture** | | | | | | |
| Prepatent pups <14d [104d (86-203d)] | | | | | | |
| Bodyweight (%) | 28 | 0.617 (0.513-0.720) | 30 | 0.434 (0.335-0.532) | -29.7% | 0.029 |
| Standard length (%) | 28 | 0.173 (0.129-0.217) | 30 | 0.213 (0.171-0.255) | +23.2% | ns |
| Pups <70cm [113d (86-203d)] | | | | | | |
| Bodyweight (%) | 56 | 0.420 (0.339-0.501) | 58 | 0.520 (0.440-0.599) | +23.8% | ns |
| Standard length (%) | 56 | 0.158 (0.132-0.184) | 58 | 0.177 (0.151-0.203) | +11.8% | ns |
| Pups ≥70cm [122d (88-196d)] | | | | | | |
| Bodyweight (%)^b^ | 35 | 0.445 (0.360-0.531) | 45 | 0.521 (0.446-0.596) | +17.2% | ns |
| Standard length (%)^b^ | 35 | 0.187 (0.154-0.221) | 45 | 0.201 (0.171-0.231) | +7.2% | ns |
|  |  |  |  |  |  |  |
| **Recruitment-to-third recapture – study duration** | | | | | | |
| Prepatent pups <14d [104d (86-203d)] | | | | | | |
| Bodyweight (%) | 28 | 0.794 (0.729-0.858) | 30 | 0.886 (0.828-0.944) | +11.4% | 0.004 |
| Standard length (%) | 28 | 0.253 (0.237-0.270) | 30 | 0.300 (0.284-0.316) | +18.4% | <0.001 |
| Pups <70cm [113d (86-203d)] | | | | | | |
| Bodyweight (%) | 56 | 0.736 (0.699-0.772) | 58 | 0.799 (0.763-0.835) | +8.6% | 0.018 |
| Standard length (%) | 56 | 0.239 (0.226-0.252) | 58 | 0.270 (0.257-0.283) | +13.0% | <0.001 |
| Pups ≥70cm [122d (88-196d)] | | | | | | |
| Bodyweight (%) | 35 | 0.595 (0.558-0.632) | 45 | 0.662 (0.629-0.694) | +11.2% | 0.010 |
| Standard length (%) | 35 | 0.205 (0.192-0.218) | 45 | 0.224 (0.213-0.236) | +9.5% | 0.033 |

^a^ Effect represents the relative difference (as a percentage) in the SGR of the treatment cohort relative to control cohort.

^b^ The final model includes a significant interaction between treatment and follow-up period length in days. The reported estimates correspond to the SGR for each treatment group at the median of follow-up period length.

^c^ The final model includes a significant interaction between treatment and pup age in days at the start of the period. The reported estimates correspond to the SGR for each treatment group at the median pup age.

ns: not significant.

# SUPPLEMENTARY TABLE S2 Comparison of predicted mean or median values (and 95% confidence intervals) of hematological parameters for treatment and control groups, across all age cohorts and for all captures of Australian sea lion pups. PCV: packed cell volume; RBC: total red blood cell count; WBC: total white blood cell count; nRBC: nucleated cell blood cell count

|  |  | **Recruitment** | | | | |  | **First recapture** | | | | |  | **Second recapture** | | | | |  | **Third recapture** | | | | |  |
| --- | --- | --- | --- | --- | --- | --- | --- | --- | --- | --- | --- | --- | --- | --- | --- | --- | --- | --- | --- | --- | --- | --- | --- | --- | --- |
|  |  | **n** | **Control** | **n** | **Treatment** | ***p*-value** |  | **n** | **Control** | **n** | **Treatment** | ***p-*value** |  | **n** | **Control** | **n** | **Treatment** | ***p-*value** |  | **n** | **Control** | **n** | **Treatment** | ***p-*value** |  |
|  | **Prepatent pups <14d old** |  |  |  |  |  |  |  |  |  |  |  |  |  |  |  |  |  |  |  |  |  |  |  |  |
|  | PCV (L/L) | 43 | 0.401 (0.385-0.417) | 45 | 0.403 (0.388-0.419) | ns |  | 36 | 0.336 (0.324-0.348) | 36 | 0.360 (0.348-0.372) | 0.011 |  | 33 | 0.384 (0.369-0.399)^d^ | 39 | 0.354 (0.340-0.368)^b^ | 0.006 |  | 27 | 0.393 (0.381-0.406)^a^ | 31 | 0.382 (0.369-0.394)^a^ | ns |  |
|  | Total plasma protein (g/L) | 42 | 64.5 (62.4-66.6)^a^ | 45 | 64.1 (62.1-66.1)^a^ | ns |  | 36 | 62.9 (61.2-64.6)^c^ | 36 | 65.5 (64.0-66.9)^c^ | 0.023 |  | 33 | 72.9 (71.5-74.3) | 39 | 66.5 (65.2-67.8) | <0.001 |  | 27 | 67.6 (66.0-69.2)^a^ | 31 | 68.4 (67.0-69.9)^a^ | ns |  |
|  | RBC (x1012/L) | 40 | 4.12 (3.97-4.26) | 45 | 4.19 (4.06-4.32) | ns |  | 34 | 3.70 (3.59-3.81) | 34 | 3.89 (3.78-4.00) | 0.016 |  | 33 | 4.17 (4.04-4.29) | 36 | 4.10 (3.98-4.21) | ns |  | 27 | 4.46 (4.31-4.62) | 29 | 4.43 (4.29-4.58) | ns |  |
|  | Haemoglobin (g/L) | 40 | 140.3 (134.6-146.0) | 45 | 142.9 (137.5-148.3) | ns |  | 34 | 120.0 (116.2-123.5)^a^ | 34 | 126.4 (123.0-129.6)^a^ | 0.012 |  | 33 | 134.4 (130.3-138.6)^d^ | 36 | 132.2 (128.3-136.1)^b^ | ns |  | 27 | 141.9 (136.8-146.8)^a^ | 29 | 143.4 (138.5-148)^a^ | ns |  |
|  | Platelets (109/L) | 38 | 318.0 (275.9-360.2) | 43 | 309.7 (270.1-349.3) | ns |  | 32 | 416.7 (364.2-469.2) | 33 | 404.8 (353.1-456.5) | ns |  | 32 | 440.3 (399.7-485.1)^a^ | 36 | 394.3 (360.0-431.9)^a^ | ns |  | 27 | 405.3 (354.7-455.8) | 29 | 433.2 (384.5-482.0) | ns |  |
|  | WBC (109/L) | 39 | 11.36 (10.30-12.54)^ab^ | 44 | 10.78 (9.78-11.87)^ab^ | ns |  | 34 | 10.02 (9.34-10.74)^ad^ | 33 | 9.57 (8.93-10.26)^ad^ | ns |  | 33 | 9.53 (8.56-10.60)^a^ | 36 | 9.53 (8.60-10.55)^a^ | ns |  | 27 | 8.45 (7.77-9.26)^a^ | 29 | 8.78 (8.08-9.62)^a^ | ns |  |
|  | Neutrophils (109/L) | 39 | 8.04 (6.91-9.35)^ab^ | 44 | 6.12 (5.28-7.09)^ab^ | 0.012 |  | 34 | 5.76 (5.17-6.35) | 33 | 6.07 (5.47-6.67) | ns |  | 33 | 5.54 (4.82-6.36)^a^ | 36 | 5.72 (5.01-6.54)^a^ | ns |  | 27 | 5.58 (4.71-6.62)^a^ | 29 | 4.75 (4.03-5.60)^a^ | ns |  |
|  | Lymphocytes (109/L) | 40 | 2.94 (2.65-3.27)^a^ | 44 | 2.84 (2.57-3.14)^a^ | ns |  | 34 | 2.85 (2.58-3.13) | 33 | 2.71 (2.43-2.98) | ns |  | 33 | 2.49 (2.16-2.87)^a^ | 36 | 2.68 (2.34-3.08)^a^ | ns |  | 27 | 2.28 (1.98-2.64)^a^ | 29 | 2.67 (2.32-3.06)^a^ | ns |  |
|  | Monocytes (109/L) | 40 | 0.59 (0.46-0.75)^ab^ | 44 | 0.49 (0.37-0.63)^as^ | ns |  | 34 | 0.60 (0.50-0.70) | 33 | 0.63 (0.53-0.73) | ns |  | 33 | 0.48 (0.39-0.59)^a^ | 36 | 0.41 (0.32-0.50)^a^ | ns |  | 27 | 0.43 (0.36-0.51) | 29 | 0.44 (0.37-0.51) | ns |  |
|  | Eosinophils (109/L) | 40 | 0.38 (0.26-0.53)^a^ | 44 | 0.44 (0.31-0.59)^a^ | ns |  | 34 | 1.22 (1.02-1.44)^a^ | 33 | 0.29 (0.20-0.40)^a^ | <0.001 |  | 33 | 0.62 (0.43-0.87)^a^ | 36 | 0.32 (0.21-0.48)^a^ | 0.037 |  | 27 | 0.28 (0.18-0.41)^a^ | 29 | 0.36 (0.24-0.50)^a^ | ns |  |
|  | Reticulocytes (10^9^/L) | 39 | 19.3 (13.9-25.9)^ac^ | 43 | 15.5 (11.0-20.9)^ac^ | ns |  | 32 | 64.1 (51.3-78.3)^a^ | 33 | 49.1 (38.1-61.4)^a^ | ns |  | 33 | 51.3 (41.8-63.0)^a^ | 36 | 48.9 (40.2-59.5)^a^ | ns |  | 27 | 45.7 (35.4-56.70)^a^ | 29 | 45.7 (35.9-56.40)^a^ | ns |  |
|  | nRBC (10^6/L) | 40 | 0.000 (0.000-0.000)^a^ | 44 | 0.000 (0.000-0.000)^a^ | ns |  | 33 | 2.056 (0.077-34.396)^a^ | 33 | 0.003 (0.000-0.125)^a^ | 0.012 |  | 33 | 0.000 (0.000-0.048)^ab^ | 36 | 0.000 (0.000-0.076)^as^ | ns |  | 27 | 0.000 (0.000-0.000)^a^ | 29 | 0.000 (0.000-0.000)^a^ | 0.026 |  |
|  |  |  |  |  |  |  |  |  |  |  |  |  |  |  |  |  |  |  |  |  |  |  |  |  |  |
|  | **Pups <70cm standard length** | |  |  |  |  |  |  |  |  |  |  |  |  |  |  |  |  |  |  |  |  |  |  |  |
|  | PCV (L/L) | 89 | 0.363 (0.353-0.374) | 92 | 0.365 (0.354-0.375) | ns |  | 72 | 0.344 (0.335-0.352) | 78 | 0.357 (0.349-0.365) | 0.03 |  | 64 | 0.382 (0.373-0.391) | 76 | 0.368 (0.359-0.376) | 0.031 |  | 53 | 0.395 (0.386-0.405)^a^ | 58 | 0.387 (0.378-0.396)^a^ | ns |  |
|  | Total plasma protein (g/L) | 89 | 61.2 (59.8-62.5) | 91 | 60.8 (59.4-62.1) | ns |  | 72 | 66.9 (65.7-68.1) | 77 | 66.9 (65.7-68.1) | ns |  | 64 | 72.2 (71.2-73.2)^a^ | 76 | 67.2 (66.4-68.0)^a^ | <0.001 |  | 53 | 69.1 (67.9-70.2)^a^ | 58 | 68.8 (67.7-69.8)^a^ | ns |  |
|  | RBC (x1012/L) | 83 | 3.81 (3.71-3.91) | 89 | 3.83 (3.73-3.93) | ns |  | 69 | 3.81 (3.72-3.90) | 74 | 3.89 (3.80-3.97) | ns |  | 63 | 4.27 (4.19-4.35) | 72 | 4.23 (4.16-4.30) | ns |  | 52 | 4.53 (4.42-4.63)^a^ | 54 | 4.51 (4.40-4.61)^a^ | ns |  |
|  | Haemoglobin (g/L) | 83 | 128.7 (124.9-132.4) | 89 | 129.2 (125.6-132.8) | ns |  | 69 | 120.6 (117.7-123.5)^d^ | 74 | 126 (123.1-128.8)^d^ | 0.01 |  | 63 | 134.9 (132.0-137.9)^d^ | 72 | 135.0 (132.2-137.7)^b^ | ns |  | 52 | 142.7 (139.1-146.3)^a^ | 54 | 142.4 (138.8-145.9)^a^ | ns |  |
|  | Platelets (109/L) | 80 | 345.3 (312.1-379.7)^a^ | 86 | 345.5 (313.4-378.6)^a^ | ns |  | 64 | 413.2 (371.8-454.7) | 72 | 407.2 (368.2-446.2) | ns |  | 60 | 418.0 (388.9-447.1) | 73 | 412.6 (386.3-439.0) | ns |  | 51 | 402.9 (371.3-433.3)^a^ | 55 | 405.4 (375.1-434.6)^a^ | ns |  |
|  | WBC (109/L) | 82 | 12.22 (11.45-13.05)^a^ | 88 | 12.33 (11.58-13.14)^a^ | ns |  | 69 | 10.48 (9.87-11.13)^a^ | 72 | 9.86 (9.30-10.46)^a^ | ns |  | 63 | 9.01 (8.49-9.59)^a^ | 72 | 8.90 (8.43-9.43)^a^ | ns |  | 52 | 8.95 (8.38-9.61)^a^ | 55 | 8.79 (8.26-9.41)^a^ | ns |  |
|  | Neutrophils (109/L) | 82 | 6.71 (6.04-7.46)^a^ | 88 | 6.73 (6.08-7.46)^a^ | ns |  | 69 | 5.38 (4.95-5.86)^ac^ | 72 | 6.26 (5.77-6.78)^ac^ | 0.012 |  | 63 | 5.34 (4.87-5.84)^a^ | 72 | 5.44 (5.00-5.92)^a^ | ns |  | 52 | 5.32 (4.74-5.98)^a^ | 55 | 5.11 (4.56-5.72)^a^ | ns |  |
|  | Lymphocytes (109/L) | 83 | 3.25 (3.01-3.5)^a^ | 89 | 3.31 (3.08-3.55)^a^ | ns |  | 69 | 3.15 (2.88-3.43)^a^ | 74 | 2.60 (2.37-2.84)^a^ | 0.006 |  | 63 | 2.64 (2.38-2.92)^a^ | 73 | 2.65 (2.41-2.91)^a^ | ns |  | 51 | 2.61 (2.35-2.90)^a^ | 55 | 2.73 (2.46-3.02)^a^ | ns |  |
|  | Monocytes (109/L) | 82 | 0.59 (0.52-0.67)^a^ | 89 | 0.62 (0.55-0.70)^a^ | ns |  | 68 | 0.51 (0.45-0.58)^a^ | 74 | 0.56 (0.50-0.63)^a^ | ns |  | 63 | 0.44 (0.37-0.51)^a^ | 73 | 0.49 (0.43-0.56)^a^ | ns |  | 52 | 0.42 (0.36-0.50)^a^ | 55 | 0.48 (0.41-0.56)^a^ | ns |  |
|  | Eosinophils (109/L) | 82 | 0.85 (0.71-1.01)^a^ | 88 | 0.91 (0.76-1.07)^a^ | ns |  | 69 | 0.86 (0.65-1.13)^ad^ | 73 | 0.30 (0.20-0.42)^ad^ | <0.001 |  | 63 | 0.51 (0.38-0.66)^a^ | 73 | 0.30 (0.22-0.40)^a^ | 0.034 |  | 52 | 0.29 (0.21-0.38)^a^ | 55 | 0.29 (0.22-0.38)^a^ | ns |  |
|  | Reticulocytes (109/L) | 79 | 33.5 (26.9-41.2)^ac^ | 88 | 25.7 (20.8-31.5)^ac^ | ns |  | 65 | 60.5 (48.0-74.5)^a^ | 74 | 65.8 (53.9-78.9)^a^ | ns |  | 63 | 58.9 (50.9-67.5)^a^ | 73 | 46.0 (39.5-53.1)^a^ | 0.022 |  | 52 | 41.6 (32.9-51.2)^ab^ | 55 | 34.2 (26.6-42.8)^ab^ | ns |  |
|  | nRBC (10^6/L) | 81 | 0.006 (0-0.188)^ab^ | 86 | 0.000 (0.000-0.001)^ab^ | 0.019 |  | 66 | 2.531 (0.287-18.01)^a^ | 71 | 0.008 (0.000-0.094)^a^ | 0.001 |  | 63 | 0.000 (0.000-0.000)^ac^ | 73 | 0.000 (0.000-0.000)^ac^ | ns |  | 52 | 0.000 (0.000-0.000)^a^ | 55 | 0.000 (0.000-0.000)^a^ | ns |  |
|  |  |  |  |  |  |  |  |  |  |  |  |  |  |  |  |  |  |  |  |  |  |  |  |  |  |
|  | **Pups ≥70cm standard length** | |  |  |  |  |  |  |  |  |  |  |  |  |  |  |  |  |  |  |  |  |  |  |  |
|  | PCV (L/L) | 60 | 0.346 (0.336-0.356)^a^ | 58 | 0.341 (0.331-0.351)^a^ | ns |  | 54 | 0.376 (0.362-0.389)^ad^ | 56 | 0.356 (0.341-2.802)^ad^ | ns |  | 50 | 0.393 (0.385-0.400)^a^ | 54 | 0.389 (0.381-0.396)^a^ | ns |  | 35 | 0.406 (0.396-0.416)^a^ | 45 | 0.407 (0.398-0.415)^a^ | ns |  |
|  | Total plasma protein (g/L) | 60 | 63.3 (61.8-65.0)^a^ | 58 | 62.8 (61.3-64.5)^a^ | ns |  | 54 | 70.8 (69.4-72.2)^d^ | 56 | 69.7 (68.4-71.1)^d^ | ns |  | 50 | 73.1 (71.9-74.3)^ad^ | 53 | 69.2 (68.2-70.3)^ab^ | <0.001 |  | 35 | 70.8 (69.2-72.4)^a^ | 44 | 69.7 (68.3-71.1)^a^ | ns |  |
|  | RBC (x1012/L) | 57 | 3.70 (3.60-3.80) | 55 | 3.67 (3.57-3.77) | ns |  | 51 | 3.87 (3.79-3.95) | 51 | 4.08 (3.99-4.16) | 0.001 |  | 50 | 4.41 (4.32-4.50)^a^ | 50 | 4.41 (4.31-4.50)^a^ | ns |  | 33 | 4.78 (4.65-4.91) | 41 | 4.63 (4.51-4.75) | ns |  |
|  | Haemoglobin (g/L) | 57 | 123.9 (120.4-127.5) | 55 | 117.8 (114.2-121.5) | 0.020 |  | 51 | 124.5 (121.3-127.6)^a^ | 51 | 131.7 (128.8-134.6)^a^ | 0.001 |  | 50 | 139.4 (136.2-142.6)^a^ | 51 | 139.8 (136.6-142.9)^a^ | ns |  | 34 | 149.7 (144.6-154.7) | 42 | 144.8 (140.3-149.3) | ns |  |
|  | Platelets (109/L) | 57 | 420.2 (381.7-458.8) | 54 | 439.5 (399.9-479.1) | ns |  | 51 | 498.9 (468.3-529.5) | 50 | 392.7 (361.8-423.6) | <0.001 |  | 50 | 433.0 (396.6-469.3) | 50 | 358.2 (321.8-394.5) | 0.008 |  | 33 | 401.2 (361.7-440.6) | 41 | 405.5 (370.3-440.7) | ns |  |
|  | WBC (109/L) | 56 | 11.93 (11.09-12.83)^a^ | 54 | 11.63 (10.80-12.53)^a^ | ns |  | 50 | 11.28 (10.52-12.09)^a^ | 50 | 9.87 (9.21-10.58)^a^ | 0.009 |  | 50 | 9.79 (9.04-10.60)^a^ | 51 | 9.01 (8.33-9.75)^a^ | ns |  | 34 | 8.99 (8.29-9.81)^a^ | 42 | 9.33 (8.65-10.12)^a^ | ns |  |
|  | Neutrophils (109/L) | 57 | 6.05 (5.42-6.75)^a^ | 54 | 5.55 (4.96-6.21)^a^ | ns |  | 51 | 6.19 (5.56-6.85)^a^ | 50 | 6.06 (5.43-6.73)^a^ | ns |  | 50 | 5.51 (4.89-6.22)^a^ | 51 | 4.90 (4.35-5.53)^a^ | ns |  | 34 | 5.05 (4.51-5.70)^a^ | 42 | 5.09 (4.59-5.68)^a^ | ns |  |
|  | Lymphocytes (109/L) | 57 | 3.56 (3.25-3.89)^a^ | 54 | 3.38 (3.08-3.71)^a^ | ns |  | 50 | 2.96 (2.69-3.26)^a^ | 50 | 2.76 (2.51-3.03)^a^ | ns |  | 49 | 3.04 (2.74-3.37)^a^ | 51 | 2.57 (2.32-2.84)^a^ | 0.022 |  | 34 | 3.01 (2.67-3.39)^a^ | 42 | 3.02 (2.72-3.36)^a^ | ns |  |
|  | Monocytes (109/L) | 57 | 0.64 (0.55-0.73)^a^ | 53 | 0.58 (0.50-0.67)^a^ | ns |  | 51 | 0.63 (0.54-0.72)^a^ | 49 | 0.56 (0.48-0.64)^a^ | ns |  | 50 | 0.48 (0.40-0.56)^a^ | 51 | 0.46 (0.38-0.54)^a^ | ns |  | 34 | 0.49 (0.36-0.63)^as^ | 42 | 0.66 (0.52-0.80)^as^ | ns |  |
|  | Eosinophils (109/L) | 56 | 1.32 (1.12-1.54)^a^ | 52 | 1.49 (1.26-1.74)^a^ | ns |  | 50 | 1.18 (0.95-1.44)^a^ | 48 | 0.26 (0.18-0.37)^a^ | <0.001 |  | 50 | 0.38 (0.27-0.52)^ac^ | 51 | 0.40 (0.29-0.54)^ac^ | ns |  | 34 | 0.32 (0.22-0.42)^a^ | 42 | 0.35 (0.26-0.45)^a^ | ns |  |
|  | Reticulocytes (109/L) | 55 | 63.9 (54.0-74.7)^a^ | 54 | 72.6 (61.9-84.1)^a^ | ns |  | 49 | 66.1 (59.1-73.2) | 50 | 51.3 (44.3-58.3) | 0.004 |  | 50 | 58.3 (48.6-69.4)^a^ | 51 | 46.5 (38.4-55.7)^a^ | ns |  | 33 | 41.0 (32.5-50.6)^a^ | 42 | 45.0 (36.9-53.8)^a^ | ns |  |
|  | nRBC (10^6/L) | 57 | 30.422 (9.814-80.005)^ae^ | 52 | 83.021 (28.713-207.422)^ae^ | ns |  | 51 | 0.001 (0-0.044)^a^ | 49 | 0.000 (0.000-0.018)^a^ | ns |  | 50 | 0.000 (0.000-0.000)^a^ | 50 | 0.000 (0.000-0.000)^a^ | ns |  | 34 | 0.000 (0.000-0.000)^a^ | 41 | 0.000 (0.000-0.000)^a^ | 0.047 |  |
|  |  |  |  |  |  |  |  |  |  |  |  |  |  |  |  |  |  |  |  |  |  |  |  |  |  |

^a^ Estimates should be interpreted as medians rather than means as transformed model estimates and their associated 95% CI boundaries were back transformed prior to reporting.

^b^ Interaction of season with treatment effect in final model.

^c^ Interaction of pup age at capture with treatment effect in final model.

^d^ Interaction of baseline hematological parameter with treatment effect in final model.

^e^ Interaction of other parameter with treatment effect in final model.

References

Lindsay, S.A., Caraguel, C.G.B., and Gray, R. (2021). Topical ivermectin is a highly effective seal 'spot-on': A randomised trial of hookworm and lice treatment in the endangered Australian sea lion (*Neophoca cinerea*). *Int J Parasitol Parasites Wildl* 16**,** 275-284. doi: 10.1016/j.ijppaw.2021.11.002.
